# Responsible Clinical AI in Dentistry: Trust, Professional Autonomy, and Perceptions of Accountability Across Stakeholders in Romania—A Multidisciplinary Cross-Sectional Survey

**DOI:** 10.3390/healthcare14183003

**Published:** 2026-09-14

**Authors:** Tamara Mihut, Mihaela Pantea, Razvan Daniel Chivu, Dana-Maria Popescu-Spineni, Corina Marilena Cristache

**Affiliations:** 1 Doctoral School, “Carol Davila” University of Medicine and Pharmacy, 37 Dionisie Lupu street, 020021 Bucharest, Romania; tamara.mihut@drd.umfcd.ro; 2 Department of Prosthetic Dentistry, Faculty of Dentistry, “Carol Davila” University of Medicine and Pharmacy, 19 Plevnei Ave., 010221 Bucharest, Romania; mihaela.pantea@umfcd.ro; 3 Department of Social Medicine, Faculty of Midwifery and Nursing, “Carol Davila” University of Medicine and Pharmacy, 020021 Bucharest, Romania; razvan.chivu@umfcd.ro (R.D.C.); spineni.popescu@umfcd.ro (D.-M.P.-S.); 4 Department of Biomedical Anthropology, “Francisc Reiner” Institute of Anthropology of the Romanian Academy, 050711 Bucharest, Romania; 5 Department of Dental Techniques, “Carol Davila” University of Medicine and Pharmacy, 8, Eroii Sanitari Blvd., 050474 Bucharest, Romania

**Keywords:** artificial intelligence, dentistry, trustworthy AI, professional accountability, clinical governance, AI literacy, EU AI Act, multidisciplinary cross-sectional survey

## Abstract

**Highlights:**

**What are the main findings?**

- Across 287 dental, legal, forensic, and healthcare stakeholders in Romania, cross-professional consensus on ethical and governance safeguards for clinical Artificial Intelligence (AI) was strong (84–91%), spanning testing on diverse populations, independent legal auditing, patient disclosure, and refusal to trust systems whose outputs cannot be verified.
- Perceived accountability for AI-related malpractice extended beyond the individual clinician: most respondents assigned it to the treating clinician (77.4%), but many also extended it to developers (49.5%) and healthcare institutions (28.3%). In contrast, awareness of the EU AI Act (32.8%) and AI-educational exposure (25.2%) remained limited.

**What are the implications of the main findings?**

- Clear normative expectations contrasted with limited regulatory preparedness, supporting stronger AI education and clearer accountability frameworks, while certification remained a specific stakeholder preference.
- The findings indicate that stakeholders perceive responsibility for clinical AI as extending across clinicians, developers, and healthcare institutions, instead of one resting exclusively on the individual practitioner.

**Abstract:**

**Background/Objectives:** Artificial intelligence (AI) is entering clinical dentistry more quickly than the professional, ethical, and regulatory frameworks intended to govern it. Responsible implementation depends not only on diagnostic performance but on trust, professional autonomy, and clear accountability—dimensions rarely examined across the different stakeholder groups who shape dental care. This study assessed perceptions of trustworthy AI, accountability, and regulatory preparedness among dental and non-dental stakeholders in Romania. **Methods:** A multidisciplinary cross-sectional survey using purposive, non-probability sampling, reported in accordance with the CROSS guideline, was administered to dental professionals, legal professionals, forensic physicians, and other healthcare stakeholders. The instrument addressed AI literacy, governance and accountability, ethics and transparency, and clinical implementation. Responses were compared across professional groups using chi-square and Kruskal–Wallis tests with Benjamini–Hochberg or Holm correction, and the questionnaire structure was examined through an exploratory psychometric assessment. Of 304 submissions, 287 were retained for analysis. **Results:** Agreement on ethical and governance safeguards was high (84–91%), including testing on diverse populations, independent auditing, and patient disclosure. Between-group differences concentrated in AI awareness, literacy, and educational exposure, as well as in governance and accountability, whereas ethics and transparency items showed broad consensus. Respondents attributed responsibility for AI-related outcomes to clinicians (77.4%), developers (49.5%), and healthcare institutions (28.3%). Only a minority reported AI Act awareness (32.8%) or previous participation in AI-related courses or conferences (25.2%). **Conclusions:** Romanian stakeholders included in this multidisciplinary sample share strong normative expectations for trustworthy clinical AI but report limited regulatory awareness and educational exposure. These findings support the development of structured AI preparedness initiatives and clearer governance frameworks reflecting stakeholders’ perceptions of responsibility as extending across clinicians, developers, and healthcare institutions. Professional certification before clinical AI use emerged as a specific stakeholder preference.

## 1. Introduction

Artificial intelligence (AI) is rapidly progressing from experimental development to clinically applicable systems capable of supporting diagnosis, treatment planning, image interpretation, patient monitoring, and other components of healthcare delivery. Dentistry has been particularly receptive to these developments because of its extensive use of digital imaging, structured clinical data, and increasingly digital diagnostic and therapeutic workflows [1,2,3]. As AI systems become more closely integrated into clinical practice, however, the central question is no longer solely whether they can generate accurate or useful outputs, but under what conditions clinicians can responsibly rely on those outputs while preserving clinical reasoning, professional autonomy, patient safety, and accountability [4].

This transition from technological feasibility to responsible clinical implementation has placed the ethical and governance dimensions of AI at the centre of contemporary healthcare debate. International and European frameworks—including guidance from the World Health Organization, the European Union Artificial Intelligence Act, the General Data Protection Regulation (GDPR), and policy statements issued by the FDI World Dental Federation—emphasize transparency, human oversight, accountability, fairness, data protection, and appropriate governance throughout the lifecycle of clinical AI systems [5,6,7,8]. Collectively, these frameworks reinforce the principle that technological performance alone is insufficient to justify clinical deployment. AI systems must also operate within organizational and professional environments capable of ensuring responsible human oversight and protecting patients’ rights.

These considerations are particularly important when AI contributes to clinical reasoning and decision-making. An AI recommendation that cannot be adequately understood or critically appraised may challenge the clinician’s ability to exercise independent professional judgement, even when formal responsibility for the clinical decision remains human [9,10,11]. Similarly, the use of AI in diagnosis or treatment raises questions concerning whether and how patients should be informed of algorithmic involvement, how meaningful consent should be obtained when AI systems access or process health data, and how patient trust may be affected by automated decision support [12,13]. Algorithmic performance that varies across demographic or clinical populations introduces an additional concern related to fairness and healthcare justice, particularly when training data are insufficiently representative. Finally, when an AI-assisted decision contributes to an adverse outcome, determining the respective responsibilities of clinicians, developers, healthcare institutions, and other actors becomes increasingly complex [14,15,16]. Thus, the ethical challenges associated with clinical AI encompass not only algorithmic transparency, but also professional autonomy, informed consent, responsibility, equity, and the preservation of the clinician–patient relationship [17].

Despite growing attention to these issues, empirical evidence regarding how stakeholders understand the responsible integration of AI in dentistry remains limited [15,18,19]. Existing studies have predominantly focused on dentists, dental students, and postgraduate trainees, generally examining AI knowledge, familiarity, perceived usefulness, attitudes, and willingness to adopt emerging technologies [18,20,21,22]. Some studies have reported broadly favourable attitudes towards AI, whereas others have identified limited familiarity, uncertainty, and skepticism regarding its clinical use [13,18,19,21,22,23]. Across these studies, however, recurrent concerns include transparency, explainability, legal accountability, data protection, algorithmic bias, and the preservation of professional judgement [11,13,16,23]. Although this literature provides important information regarding clinicians’ preparedness for AI-assisted practice, it captures only one component of the broader ethical and institutional environment in which clinical AI is implemented.

Responsible AI integration is inherently multidisciplinary because the conditions governing its use are shaped by actors extending beyond the individual clinician. Healthcare organizations determine which systems are procured and deployed and how their performance is monitored; developers influence system design, validation, and transparency; legal professionals interpret emerging questions of responsibility and liability; regulators and professional organizations establish standards of acceptable practice; and patients are directly affected by decisions concerning disclosure, consent, data governance, and access to AI-supported care [15,24,25]. Despite this inherently multidisciplinary governance environment, empirical research in dentistry has predominantly examined dentists, dental students, or other healthcare professionals in isolation. Comparisons incorporating clinical, legal, forensic, institutional, and broader healthcare perspectives remain comparatively scarce [14,15,18]. Consequently, it remains unclear whether the stakeholder groups involved in governing and implementing clinical AI share similar expectations regarding trust, accountability, patient protection, and regulatory preparedness. To our knowledge, direct cross-professional comparisons of these perspectives using a common survey instrument have not previously been reported in the context of AI-assisted dentistry.

National context is also relevant because the translation of general ethical principles and European regulatory requirements into clinical practice occurs through country-specific healthcare systems, professional regulations, institutional structures, and patterns of service delivery. Romania provides a pertinent setting in which to examine this process. As a European Union Member State, Romania operates within the regulatory framework established by the EU AI Act and the GDPR, while the practical implementation of AI in healthcare also depends on national authorities, professional organizations, educational institutions, healthcare providers, and available digital infrastructure. Dentistry represents an especially informative clinical setting because AI applications intersect directly with diagnostic imaging, treatment planning, prosthetic design, patient data processing, and clinical decision support, while implementation may occur in organizational environments with substantially different levels of technical and governance capacity. Examining how Romanian stakeholders understand these responsibilities can therefore provide context-specific evidence while contributing to the broader European discussion concerning trustworthy and ethically responsible clinical AI.

Accordingly, the primary objective of this cross-sectional study was to compare perceptions of AI-assisted dentistry among four stakeholder groups in Romania: dental professionals (Group 1), legal professionals (Group 2), forensic physicians (Group 3), and healthcare stakeholders, including healthcare administrators and patient representatives (Group 4). The study examined the ethical, professional, legal, and institutional conditions considered necessary for responsible AI integration, with particular emphasis on trust and explainability, professional autonomy and preparedness, patient information and data protection, algorithmic fairness and healthcare equity, responsibility allocation, and governance of AI-assisted clinical decision-making.

As a secondary objective, the study examined perceptions regarding the implementation of AI within public dental services and explored factors associated with these perceptions. An exploratory objective was to assess the psychometric structure of the study-specific questionnaire. By identifying areas of consensus and divergence across stakeholder groups, the study seeks to provide empirical evidence relevant to the development of ethically grounded, clinically appropriate, and institutionally sustainable approaches to AI-assisted dental care.

## 2. Materials and Methods

### 2.1. Study Design and Reporting Standards

This cross-sectional observational survey was conducted in Romania between April 2025 and April 2026 to investigate multidisciplinary stakeholder perspectives on the ethical, professional, legal, and institutional conditions required for the responsible integration of artificial intelligence (AI) into dental practice. Particular attention was given to AI-related trust and explainability, professional autonomy and responsibility, regulatory oversight, patient information and data protection, algorithmic fairness, professional preparedness, and implementation within the healthcare system.

The study adopted a multidisciplinary design involving stakeholders whose professional or societal roles may influence the development, regulation, implementation, oversight, or use of AI-assisted dental care. The study was reported in accordance with the Consensus-Based Checklist for Reporting of Survey Studies (CROSS) Supplementary Materials (Table S1) [26].

### 2.2. Questionnaire Development

Because no validated multidisciplinary instrument was identified that comprehensively addressed the ethical, legal, professional, and institutional dimensions of AI integration in dentistry, a study-specific questionnaire was developed. Questionnaire development followed a structured process comprising: (1) definition of the conceptual framework; (2) generation and organization of questionnaire items; (3) multidisciplinary expert review and pilot testing; and (4) revision and finalization of the instrument.

The conceptual framework was informed by published literature addressing AI implementation in healthcare and dentistry, together with major international ethical, professional, and regulatory frameworks, including the World Health Organization guidance on the ethics and governance of AI for health, the General Data Protection Regulation (GDPR), the European Union Artificial Intelligence Act, and policy statements issued by the FDI World Dental Federation [5,6,7,8,12]. These sources were used to identify domains relevant to the responsible clinical use of AI, including perceived clinical applications and benefits, AI literacy and professional preparedness, system validation and regulatory oversight, professional and institutional accountability, patient data protection, algorithmic fairness, transparency and explainability, professional education, and implementation within healthcare services.

The initial questionnaire was reviewed by a multidisciplinary expert panel comprising seven dental and legal professionals. Two oral and maxillofacial surgeons, one pediatric dentist, one prosthodontist, and three lawyers were purposively selected on the basis of their relevant expertise and professional experience. All four dental experts had experience with AI-enabled devices, and one was also a university professor. Each of the three legal experts had more than 20 years of professional experience in civil, criminal, administrative, and commercial law, with one also having specific expertise in medical law. The experts independently assessed the relevance, clarity, comprehensibility, and appropriateness of individual items and the overall coverage of the proposed domains. Their feedback was used to revise ambiguous or repetitive items and to improve the clarity and structure of the questionnaire. No formal Content Validity Index (CVI) was calculated. The revised questionnaire was pilot-tested in 10 participants representing the relevant professional backgrounds to assess item comprehensibility, clarity, completion time, and practical feasibility. Pilot participants were not included in the final analytical sample. Feedback obtained during pilot testing was used to refine item wording and organization and to produce the final version of the questionnaire before broader administration.

### 2.3. Survey Instrument

The final survey instrument comprised 53 items organized into 12 thematic sections and included single-choice, multiple-response, and 10-point Likert-type items. The questionnaire required approximately 10–15 min to complete. It was administered in Romanian, and the English translation is provided in the Supplementary Materials (Table S2).

The instrument collected information on electronic informed consent; demographic and professional characteristics, including age, gender, professional group, dental specialty, academic affiliation, geographical region, and practice setting; and familiarity with AI and its perceived clinical applications and benefits. Subsequent sections addressed AI testing and validation, regulatory oversight, professional and institutional responsibility, data protection, AI-related malpractice, professional guidance and education, algorithmic bias and fairness, transparency and explainability, patient information, implementation within the public healthcare system, AI-related advertising and marketing, and alignment of Romanian regulation with European and international frameworks.

For multiple-response questions, participants could select more than one predefined option, and selected questions included an open-ended “Other” option to capture responses not represented by the predefined categories. For statistical analysis, each response option from multiple-response items was treated as a separate binary variable (selected/not selected). Because multiple options could be selected by the same respondent, binary variables derived from the same multiple-response item were not statistically independent; this within-respondent dependence was acknowledged when interpreting option-level comparisons.

Responses to Likert-type items were recorded on a 10-point scale, with anchors adapted to the construct being assessed, ranging from 1 (strongly disagree/not important/not familiar) to 10 (strongly agree/very important/very familiar), as appropriate. Higher values therefore represented greater agreement, perceived importance, or familiarity. Free-text responses entered under the “Other” option were reviewed and grouped into conceptually similar categories before descriptive analysis.

### 2.4. Study Population, Eligibility, and Recruitment

Participants were recruited using purposive, non-probability sampling from predefined stakeholder groups considered relevant to the responsible implementation of AI in dentistry. These comprised dental professionals, legal professionals, forensic physicians, and healthcare stakeholders, including healthcare administrators and patient representatives. Each stakeholder group contributed a perspective informed by its professional context: clinical practice for dental professionals, regulation and liability for legal professionals, medico-legal assessment of adverse outcomes and causation for forensic physicians, and institutional and patient-related considerations for healthcare stakeholders.

Eligible participants were required to belong to one of the predefined stakeholder categories, to provide electronic informed consent, and to agree to the processing of survey data for research purposes. Additional eligibility criteria were also applied: age ≥18 years as well as professional activity in Romania.

The questionnaire was administered electronically using Google Forms.

Electronic invitations to participate were disseminated through professional organizations, institutional mailing lists, professional networks, and publicly available professional e-mail addresses. Wider dissemination was sought through relevant Romanian organizations, including the Romanian College of Dentists, Bar Associations, the College of Legal Advisers, judicial bodies, healthcare institutions, and patient advocacy organizations. Two reminder communications were sent during the recruitment period.

Participation was voluntary. To reduce the possibility of multiple participation, respondents were asked to provide an e-mail address solely for duplicate identification. When duplicate submissions were detected, only the first complete response was retained. Questionnaires with more than 20% missing responses were excluded from the analytical dataset. E-mail addresses were removed from the analytical dataset and were not used as study variables.

### 2.5. Sample Size Considerations

No formal a priori sample-size calculation was used to determine the recruitment target because the study employed purposive sampling across several stakeholder populations for which a single reliable sampling frame was not available. Recruitment therefore aimed to obtain the largest feasible multidisciplinary sample during the predefined study period. The statistical adequacy of the final analytical sample was subsequently evaluated using G*Power, version 3.1.9.7 (Heinrich Heine University Düsseldorf, Düsseldorf, Germany). For the principal binary categorical comparisons across four stakeholder groups, assuming a chi-square test with 3 degrees of freedom, α = 0.05, and 80% statistical power, the final analytical sample of 287 participants was sufficient to detect an effect size of approximately Cohen’s w = 0.195, indicating sensitivity to effects in the small-to-moderate range. This overall assessment does not imply equivalent statistical power for all subgroup or pairwise comparisons, particularly those involving the smaller forensic physician subgroup.

### 2.6. Ethical Considerations

The study was conducted in accordance with the Declaration of Helsinki and was approved by the Research Ethics Committee of “Carol Davila” University of Medicine and Pharmacy, Bucharest, Romania (approval no. 1839, 31 January 2025).

Electronic informed consent was obtained from all participants before access to the questionnaire was granted. Participation was voluntary, and respondents could discontinue completion of the questionnaire before submission.

E-mail addresses were collected exclusively for the identification of duplicate submissions and were not included in the analytical dataset. Access to identifiable information was restricted to the principal investigator for duplicate verification. Identifying information was stored separately from survey responses, and statistical analyses were performed using an anonymized dataset. No IP addresses or other direct electronic identifiers were collected.

### 2.7. Statistical Analysis

Statistical analyses were performed using IBM SPSS Statistics for Windows, version 32.0 (IBM Corp., Armonk, NY, USA) and R version 4.5.3 (R Foundation for Statistical Computing, Vienna, Austria). SPSS was used for data management, descriptive analyses, between-group comparisons, and ordinal regression modelling, whereas R was used for exploratory psychometric analyses requiring methods appropriate for ordinal and binary variables.

Categorical variables were summarized as frequencies and percentages. Continuous demographic variables were summarized using means and standard deviations and, where appropriate, medians and interquartile ranges. Because responses to the 10-point Likert-type items were treated as ordinal variables for inferential analyses, they were primarily summarized using medians and interquartile ranges. For multiple-response questions, each response option was coded and analyzed separately as a binary variable (selected vs. not selected).

Categorical outcomes were compared across the four stakeholder groups using Pearson’s chi-square test or Fisher’s exact test, as appropriate. Associations were quantified using Cramer’s V. For binary outcomes showing a significant overall between-group association, pairwise group comparisons were performed using Fisher’s exact tests, with Benjamini–Hochberg false discovery rate (FDR) adjustment of the corresponding post hoc *p* values.

Ordinal outcomes were compared across stakeholder groups using the Kruskal–Wallis test. When an overall between-group difference was identified, pairwise comparisons were performed using Dunn’s post hoc test with Holm adjustment. Effect sizes for Kruskal–Wallis comparisons were expressed as epsilon-squared (ε^2^). For outcomes proceeding to post hoc analysis, all six pairwise group comparisons were evaluated, with multiplicity adjustment applied separately for each outcome. Effect sizes were considered alongside adjusted *p* values when interpreting between-group differences, with emphasis placed on the magnitude and substantive relevance of the observed associations in addition to their statistical significance.

Given the number of questionnaire outcomes examined, the Benjamini–Hochberg FDR procedure was applied to the complete set of global tests separately within each of four prespecified analytical domains: (1) AI awareness, literacy, and professional education (Q10, 11, 12, 13, 14, 26, 30, 31, 32, 34); (2) governance, regulation, and professional accountability (Q15, 16, 17, 22, 23, 24, 25, 27, 29, 33, 37, 44, 51, 52, 53); (3) transparency, ethics, and patient protection (Q18, 19, 20, 21, 35, 36, 42, 43, 45, 49, 50); and (4) clinical and public-sector implementation (Q28, 38, 39, 40, 41, 46, 47, 48). Post hoc comparisons were interpreted only in the context of the corresponding overall analysis. FDR-adjusted *p* values are reported for global categorical and ordinal comparisons where applicable, whereas pairwise ordinal comparisons were adjusted using the Holm procedure. Complete global results, including unadjusted and FDR-adjusted *p*-values, and complete adjusted pairwise post hoc results are provided in Supplementary Tables S4 and S5, respectively; only the most relevant between-group differences are emphasized in the main text.

Missing data were not assumed to be missing completely at random. For most items, the low level of item non-response (0–3 responses) had negligible analytical impact. However, non-response to the item on current professional applications of AI (Q13) was strongly associated with professional group (see Section 4.7), and was consistent with differential applicability of the item across professional roles. Available-case analysis was therefore used without imputation, and item-specific denominators are reported throughout.

To mitigate the resulting potential for non-response error, the Q13 comparisons are interpreted as applying to stakeholders who reported on current clinical applications rather than to each group in full, and role-tailored item routing or an explicit “not applicable to my role” option is recommended for future multi-stakeholder instruments.

Separate exploratory univariable proportional-odds ordinal logistic regression models were used to examine associations between selected predictors and respondents’ perceived feasibility of implementing AI in public dental clinics within the next five years (Q39). For analytical reporting, this construct is referred to as perceived feasibility and was measured on a 10-point ordinal scale. Two governance-related variables were examined in separate models: perceived knowledge and application of General Data Protection Regulation (GDPR) requirements in current dental practice (Q19) and exposure to AI-related professional guidelines (Q26), both measured on 10-point scales. Odds ratios (ORs) and 95% confidence intervals (CIs) were estimated for each one-point increase in the corresponding predictor. An OR greater than 1 indicated higher odds of reporting a higher perceived-feasibility category. Because each predictor was evaluated in a separate model, the reported ORs were not adjusted for other covariates. Overall model fit was evaluated using the likelihood-ratio chi-square test and Nagelkerke’s pseudo-R^2^. The proportional-odds assumption was assessed using the test of parallel lines. No multiplicity correction was applied to these two exploratory regression analyses. Given the cross-sectional design, regression estimates were interpreted as associations, not as causal effects.

Statistical significance was set at *p* < 0.05.

### 2.8. Exploratory Psychometric Analysis

The psychometric properties of the study-specific questionnaire were examined exploratorily. The analysis included 28 questionnaire items considered suitable for psychometric assessment as ordinal or binary indicators. Items were eligible when they used an ordinal (1–10) or binary response format and measured a conceptually relevant construct. Purely nominal multiple-response items and open-text items were not included in the psychometric analysis. Response concentration was considered during psychometric assessment, and highly skewed items were interpreted cautiously because of their reduced discriminating variance. Conceptually reverse-worded items were recoded so that higher scores consistently reflected higher levels of the construct being measured. Further details regarding item selection are provided in Supplementary Table S7.

Internal consistency was assessed using Cronbach’s alpha and McDonald’s omega for sets of items considered to measure conceptually related constructs. Because the eligible items comprised ordinal and binary indicators, inter-item associations were estimated using a polychoric and tetrachoric correlation matrix, with polychoric correlations used for pairs involving ordinal items and tetrachoric correlations for pairs of binary items. The suitability of eligible items for factor analysis was examined using the Kaiser–Meyer–Olkin measure of sampling adequacy and Bartlett’s test of sphericity.

Exploratory factor analysis (EFA) was performed using principal-axis factoring with oblique direct oblimin rotation, allowing the underlying dimensions to be correlated. The number of retained factors was determined primarily by parallel analysis, supported by examination of the scree plot and conceptual interpretability. A preliminary confirmatory factor analysis (CFA) was then conducted to examine the fit of the factor structure identified by EFA. Because the indicators were ordinal and binary, CFA was estimated using a robust diagonally weighted least squares (DWLS) estimator. Model fit was evaluated using the chi-square statistic, the chi-square/degrees-of-freedom ratio, comparative fit index (CFI), Tucker–Lewis index (TLI), root mean square error of approximation (RMSEA), and standardized root mean square residual (SRMR).

Because EFA and CFA were conducted using the same analytical sample, the CFA was regarded as a preliminary assessment of structural fit and not as an independent validation of the questionnaire. The resulting factor labels were therefore interpreted as descriptive empirical groupings and not as established questionnaire subscales. External validation in an independent sample, preferably using a prespecified factor structure and assessment of measurement invariance across stakeholder groups, is required before the instrument can be used to derive scored subscales.

### 2.9. Sensitivity Analyses

Sensitivity analyses were performed to assess the robustness of the principal findings. These included analyses excluding the heterogeneous healthcare stakeholder group, analyses examining the influence of the relatively small forensic physician subgroup, and analyses evaluating the influence of item-level missing data. The corresponding numerical sensitivity-analysis results are reported in Supplementary Table S8.

## 3. Results

### 3.1. Participant Flow and Characteristics

**Figure 1:**
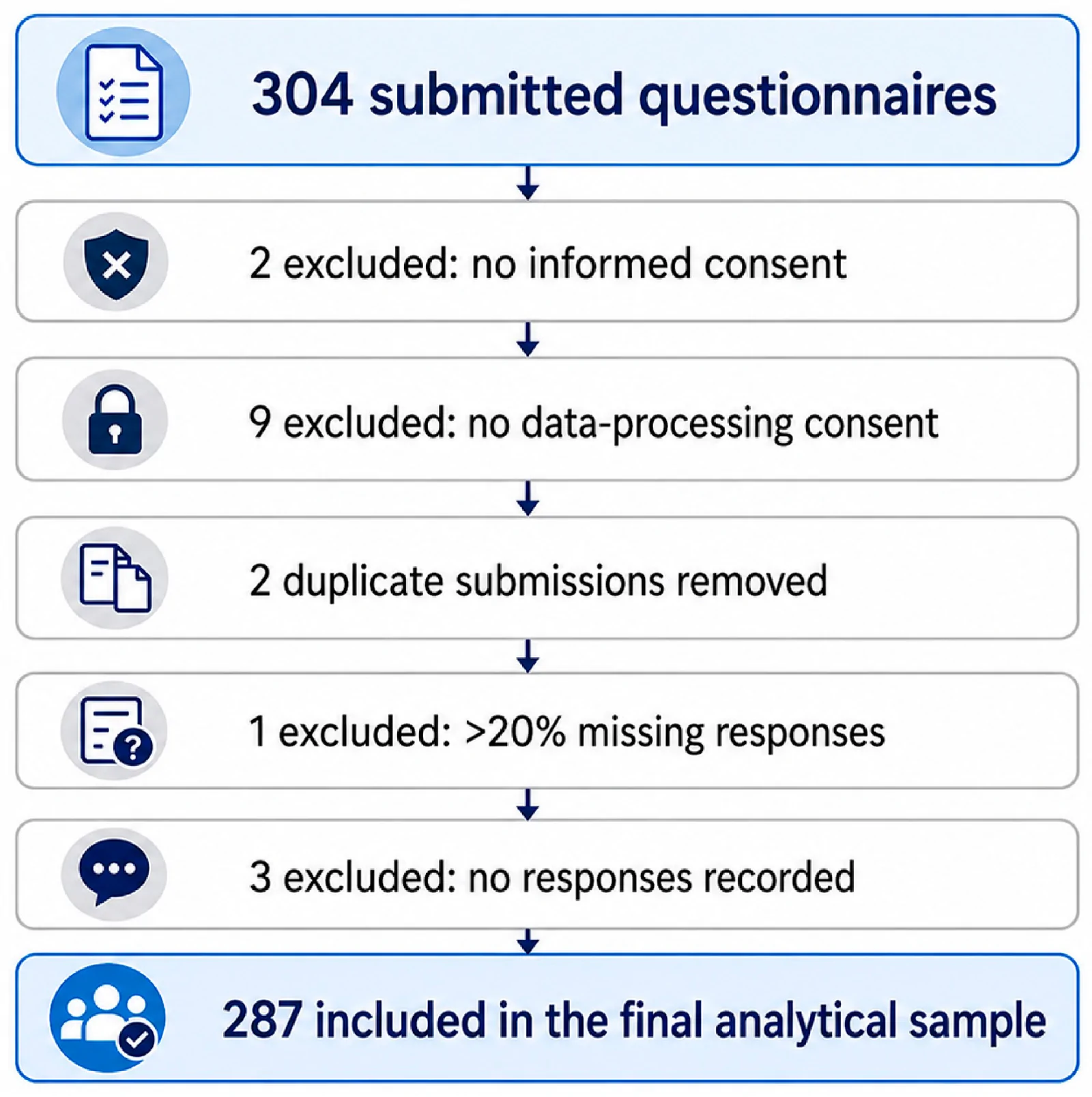
Participant flow diagram.

A total of 304 questionnaires were submitted during the study period. Of these, 17 were excluded: two because electronic informed consent was not provided, nine because consent for data processing was not granted, two because they represented duplicate submissions, one because more than 20% of questionnaire items were missing, and three because no questionnaire items had been completed, consistent with repeated submission of an empty form. The final analytical sample therefore comprised 287 participants (Figure 1). Because some survey invitations were distributed to institutions for further dissemination, the total number of individuals who received the invitation could not be determined; consequently, a conventional survey response rate could not be calculated.

**Figure 2:**
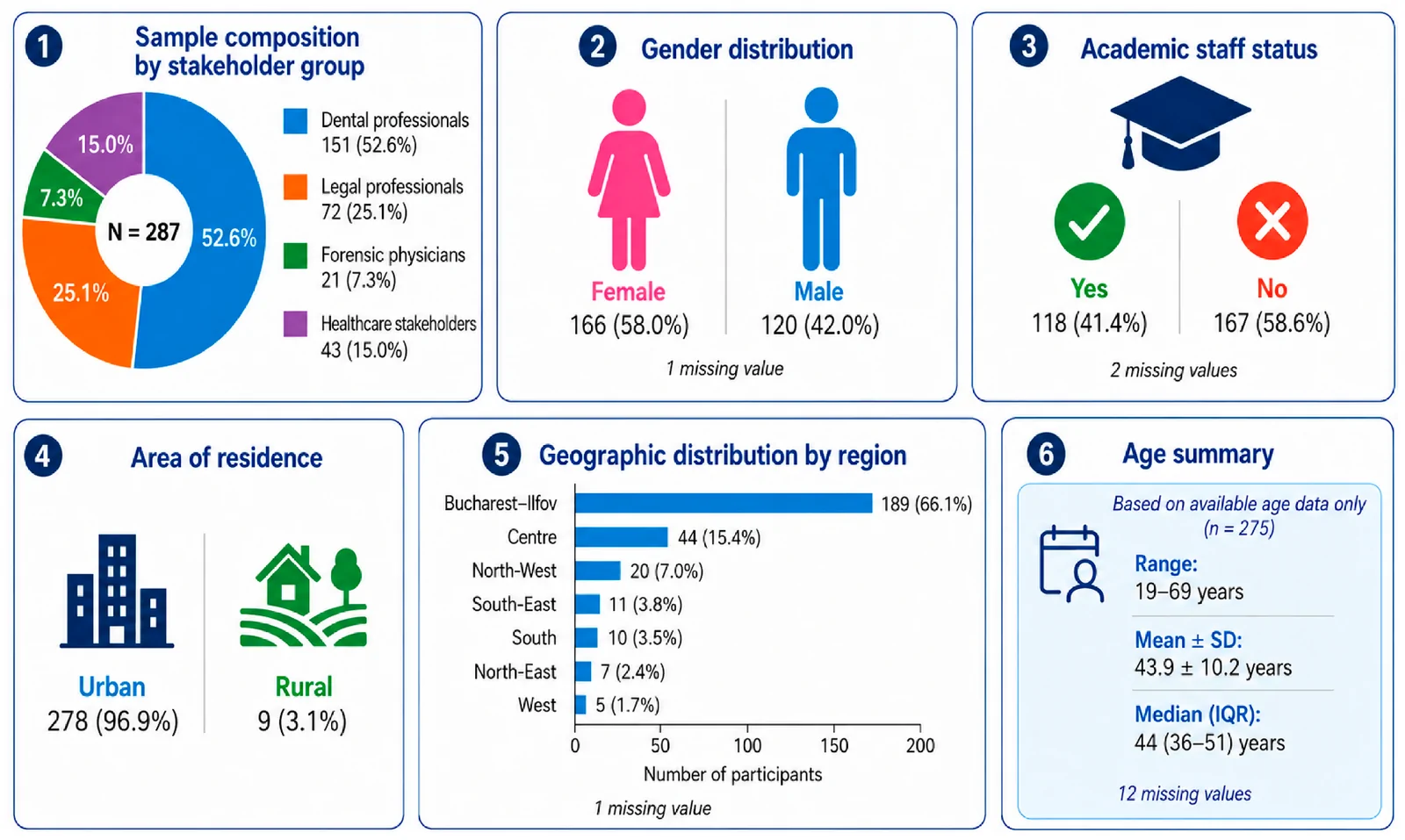
Demographic and professional characteristics of the analytical sample (n = 287). Percentages were calculated using the number of available responses for each variable. Missing values are reported separately. Means and standard deviations are provided in the Supplementary Materials (Table S3).

Among respondents providing age data (n = 275), age ranged from 19 to 69 years, with a mean of 43.9 ± 10.2 years and a median of 44 years (IQR, 36–51). Women represented 58.0% of participants with available gender data. Dental professionals constituted the largest stakeholder group (52.6%), followed by legal professionals (25.1%), healthcare stakeholders (15.0%), and forensic physicians (7.3%). The sample was predominantly urban (96.9%) and geographically concentrated in the Bucharest–Ilfov region (66.1%) (Figure 2 and Table S3).

### 3.2. Comparative Analyses by Professional Group

Across the four prespecified analytical domains, the clearest overall pattern was broad cross-stakeholder convergence on transparency, ethics, and patient-protection requirements. No between-group comparison within this domain remained statistically significant after FDR correction, whereas adjusted differences were concentrated primarily in AI awareness, literacy, and educational exposure and in selected governance and accountability items; only one adjusted difference remained within the clinical and public-sector implementation domain. The sections below therefore emphasize both the principal areas of multidisciplinary consensus and the between-group differences that persisted after multiplicity correction. Complete global and post hoc results are provided in Supplementary Tables S4 and S5.

For ease of reference, Table 1 maps the questionnaire items to the four prespecified analytical domains and provides brief item descriptions.

#### 3.2.1. AI Awareness, Literacy, and Professional Education

AI-related familiarity and educational exposure were limited across the overall sample. Fewer than half of respondents (47.4%) reported familiarity with AI-based healthcare devices (Q10), 56.1% reported basic knowledge of AI principles (Q11), and only 25.2% had previously participated in AI-related courses or conferences (Q30). Familiarity with AI testing standards was low (Q14; median, 2; IQR, 1–5), with 44.6% of respondents assigning the minimum score (Table 2). Exposure to AI-related professional guidelines was similarly limited (Q26; median, 1; IQR, 1–3). In contrast, respondents expressed strong support for the introduction of AI into undergraduate curricula (Q31; median, 8; IQR, 5–10). Hands-on workshops (48.8%) and software training (36.0%) were the educational formats selected most frequently, while 40.6% of respondents reported familiarity with the term “algorithmic bias” (Q34).

After correction for multiple comparisons, significant between-group differences remained for familiarity with AI-based healthcare devices (Q10; χ^2^(3) = 20.093, *p* < 0.001, Cramer’s V = 0.265), basic knowledge of AI principles (Q11; χ^2^(3) = 38.018, *p* < 0.001, V = 0.364), familiarity with AI testing standards (Q14; H(3) = 23.830, *p* < 0.001, ε^2^ = 0.074), exposure to AI-related professional guidelines (Q26; H(3) = 32.971, *p* < 0.001, ε^2^ = 0.107), and previous participation in AI-related courses or conferences (Q30; χ^2^(3) = 49.690, *p* < 0.001, V = 0.417) (Table 3).

For Q10, the adjusted pairwise difference remained significant between dental and legal professionals. For Q11, significant adjusted differences were identified between dental and legal professionals, dental professionals and healthcare stakeholders, legal professionals and forensic physicians, and legal professionals and healthcare stakeholders. For Q14, Dunn’s post hoc comparisons with Holm correction showed significant differences between dental and legal professionals and between dental professionals and forensic physicians. For Q26, dental professionals differed significantly from each of the other three stakeholder groups. Previous participation in AI-related courses or conferences (Q30) differed between dental and legal professionals, dental professionals and forensic physicians, dental professionals and healthcare stakeholders, and legal professionals and healthcare stakeholders (Table 3).

Responses regarding current professional applications of AI (Q13) were reported by 228 of 287 participants (79.4%); this item had the highest level of item non-response in the questionnaire (n = 59 missing), and all Q13 analyses were therefore conducted on the 228 valid responses. This non-response was unevenly distributed across professional groups (see Section 4), and the Q13 comparisons should be interpreted in that light. Adjusted between-group differences remained significant for clinical management (χ^2^(3, N = 228) = 18.719, *p* < 0.001, V = 0.287), treatment planning (χ^2^(3, N = 228) = 19.105, *p* < 0.001, V = 0.289), other applications (χ^2^(3, N = 228) = 11.162, *p* = 0.011, V = 0.221), and reporting no current AI use (χ^2^(3, N = 228) = 15.256, *p* = 0.002, V = 0.259). The corresponding significant adjusted pairwise comparisons are presented in Table 3.

At the unadjusted level, differences were also observed for the perceived benefit of AI in providing clinically relevant real-time data (Q12; χ^2^(3, N = 287) = 8.762, *p* = 0.033, V = 0.175) and for AI use in patient monitoring (Q13; χ^2^(3, N = 228) = 7.899, *p* = 0.048, V = 0.186), lesion interpretation (χ^2^(3, N = 228) = 8.385, *p* = 0.039, V = 0.192), and histopathological analysis (χ^2^(3, N = 228) = 8.383, *p* = 0.039, V = 0.192); however, none remained statistically significant after FDR adjustment.

No adjusted between-group differences were identified for support for introducing AI into undergraduate curricula (Q31), preferred educational formats (Q32), familiarity with algorithmic bias (Q34), or the remaining AI applications and perceived benefits examined in this domain. Complete global and pairwise results are provided in Supplementary Tables S4 and S5.

#### 3.2.2. Governance, Regulation, and Professional Accountability

Respondents expressed strong support for governance mechanisms intended to ensure the safe and accountable use of AI. The importance of clinical testing before implementation received a median score of 10 (IQR, 9–10; Q15), as did professional involvement in AI validation (median, 10; IQR, 8–10; Q17). The perceived importance of establishing an ethical oversight body for AI in healthcare was also high (median, 10; IQR, 7–10; Q37).

A dedicated legal framework for AI-related malpractice was supported by 74.5% of respondents (Q23), and 74.0% indicated that they would refrain from using AI if legal liability remained unclear (Q25). Standardized clinical protocols for AI-assisted dental care were supported by 89.1% (Q27). Regarding the preferred regulatory level for mandatory AI performance evaluation (Q16), 73.8% of respondents favoured combined European and national regulation, compared with 17.1% supporting European-level regulation alone and 6.6% national-level regulation alone, with the remaining 2.5% comprising other or mixed responses. Legal auditing of AI algorithms by 90.8% (Q44), and international certification standards by 89.2% (Q53). Professional certification before using AI in dental practice was supported by 63.6% (Q33), whereas only 32.8% reported awareness of the European AI Act (Q51).

Responsibility for an incorrect AI-assisted diagnosis was attributed most frequently to the clinician (77.4%), followed by the AI developer (49.5%) and the healthcare institution (28.3%) (Q22). For AI decision traceability (Q24), respondents most frequently selected clarification of responsibility between the developer and clinician (78.4%), patient information (73.5%), and documentation of training-data sources (67.2%). The Ministry of Health (75.9%), professional societies (67.8%), and universities (50.7%) were most frequently identified as responsible for developing AI clinical guidelines (Q29). For implementation of AI regulations in Romania (Q52), the Ministry of Health (93.3%), Romanian College of Dentists (83.9%), and universities (62.8%) were most frequently selected.

**Figure 3:**
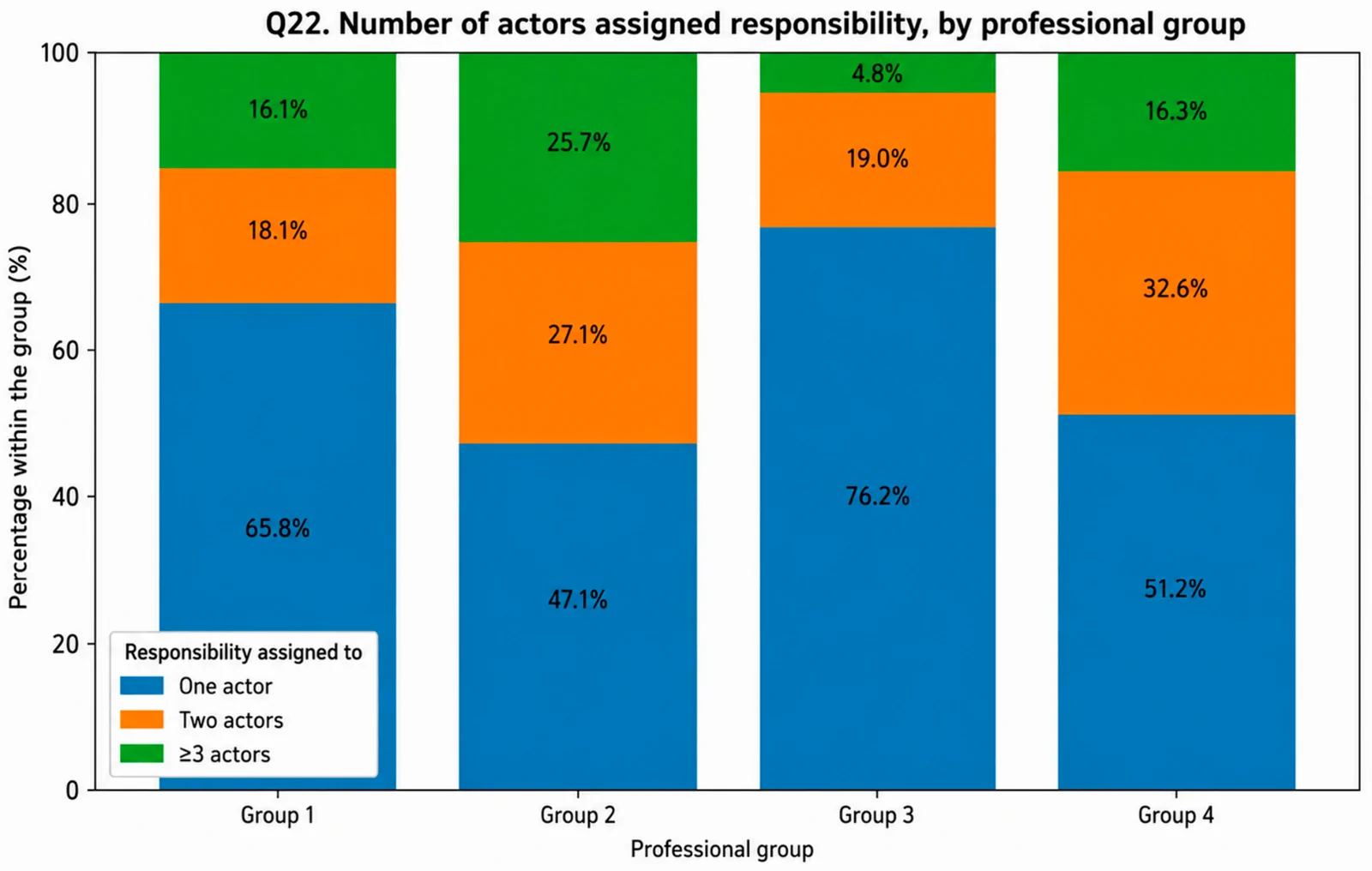
Within-group distribution of the number of actors selected by respondents as responsible for an incorrect AI-assisted diagnosis (Q22).

After FDR correction, only two between-group differences within this domain remained statistically significant. Attribution of responsibility to the healthcare institution following an incorrect AI-assisted diagnosis differed across stakeholder groups (Q22, Figure 3; χ^2^(3, N = 283) = 20.703, FDR-adjusted *p* = 0.004, V = 0.270). Institutional responsibility was selected by 20.1% of dental professionals, 45.7% of legal professionals, 9.5% of forensic physicians, and 37.2% of healthcare stakeholders. Adjusted pairwise comparisons were significant between dental and legal professionals (pFDR = 0.001) and between legal professionals and forensic physicians (pFDR = 0.006). Because the forensic physician subgroup was small (n = 21), comparisons involving this group should be interpreted cautiously.

Support for professional certification before AI use also differed across groups (Q33; χ^2^(3, N = 286) = 18.242, FDR-adjusted *p* = 0.006, V = 0.253), with support reported by 52.3% of dental professionals, 78.9% of legal professionals, 76.2% of forensic physicians, and 72.1% of healthcare stakeholders. The adjusted pairwise difference remained significant between dental and legal professionals (pFDR = 0.001).

A between-group difference also remained significant for the perceived importance of clinical testing before AI implementation (Q15; H(3) = 13.159, FDR-adjusted *p* = 0.047, ε^2^ = 0.036). Although the median score was 10 across all four groups, the distribution differed, with forensic physicians endorsing this requirement most uniformly (IQR 10–10) and legal professionals showing greater dispersion (IQR 8–10); the single significant adjusted pairwise contrast was between legal professionals and forensic physicians (Dunn’s test with Holm correction, *p* = 0.025). Given the small size of the forensic physician subgroup (n = 21), this difference should be interpreted cautiously.

Several additional unadjusted differences—including refraining from AI use when liability remained unclear (Q25), documentation of training-data sources and archiving of AI-assisted decisions (Q24), and selection of the Ministry of Justice as an implementation authority (Q52)—did not remain significant after FDR correction. No other governance or accountability outcome showed an adjusted between-group difference.

#### 3.2.3. Transparency, Ethics, and Patient Protection

Respondents consistently supported measures intended to promote transparent, ethically accountable, and equitable use of AI in dental practice.

Regarding data protection, the perceived risk of AI compromising patient data confidentiality had a median score of 5 (IQR, 2–7; Q18), while self-reported knowledge and application of the General Data Protection Regulation (GDPR) in current dental practice had a median score of 6 (IQR, 5–8; Q19). Specific legislation governing AI processing of biometric data was supported by 87.8% of respondents (Q20). Regarding AI access to electronic patient records, 61.5% supported access only with prior patient consent, 10.8% supported unrestricted access, and 27.6% opposed such access (Q21).

Explainability was strongly prioritized. The importance of AI providing clear explanations for its decisions received a median score of 10 (IQR, 8–10; Q42), with 242 respondents (84.3%) assigning scores of 8–10. Moreover, 87.8% indicated that they would not trust an AI recommendation if its underlying reasoning could not be verified (Q43). A legal obligation to inform patients when AI contributes to therapeutic decision-making was supported by 84.3% (Q45), while 73.1% supported clear labelling of automated AI applications used in patient communication (Q49).

Fairness and regulatory safeguards were also prominent. Overall, 61.2% considered that AI could negatively affect equity in dental care (Q35), while 91.3% supported testing AI systems on diverse patient populations before clinical authorization (Q36). The importance of aligning Romanian legislation with the European AI Act received a median score of 10 (IQR, 8–10; Q50).

At the unadjusted level, only the legal obligation to inform patients when AI was used in therapeutic decision-making showed a significant between-group association (Q45; χ^2^(6) = 16.409, *p* = 0.012, V = 0.169); this association did not remain significant after FDR correction (adjusted *p* = 0.075). Importantly, the between-group comparison for perceived confidentiality risk (Q18) was not statistically significant even before adjustment (H(3) = 7.624, *p* = 0.054, ε^2^ = 0.016).

**Figure 4:**
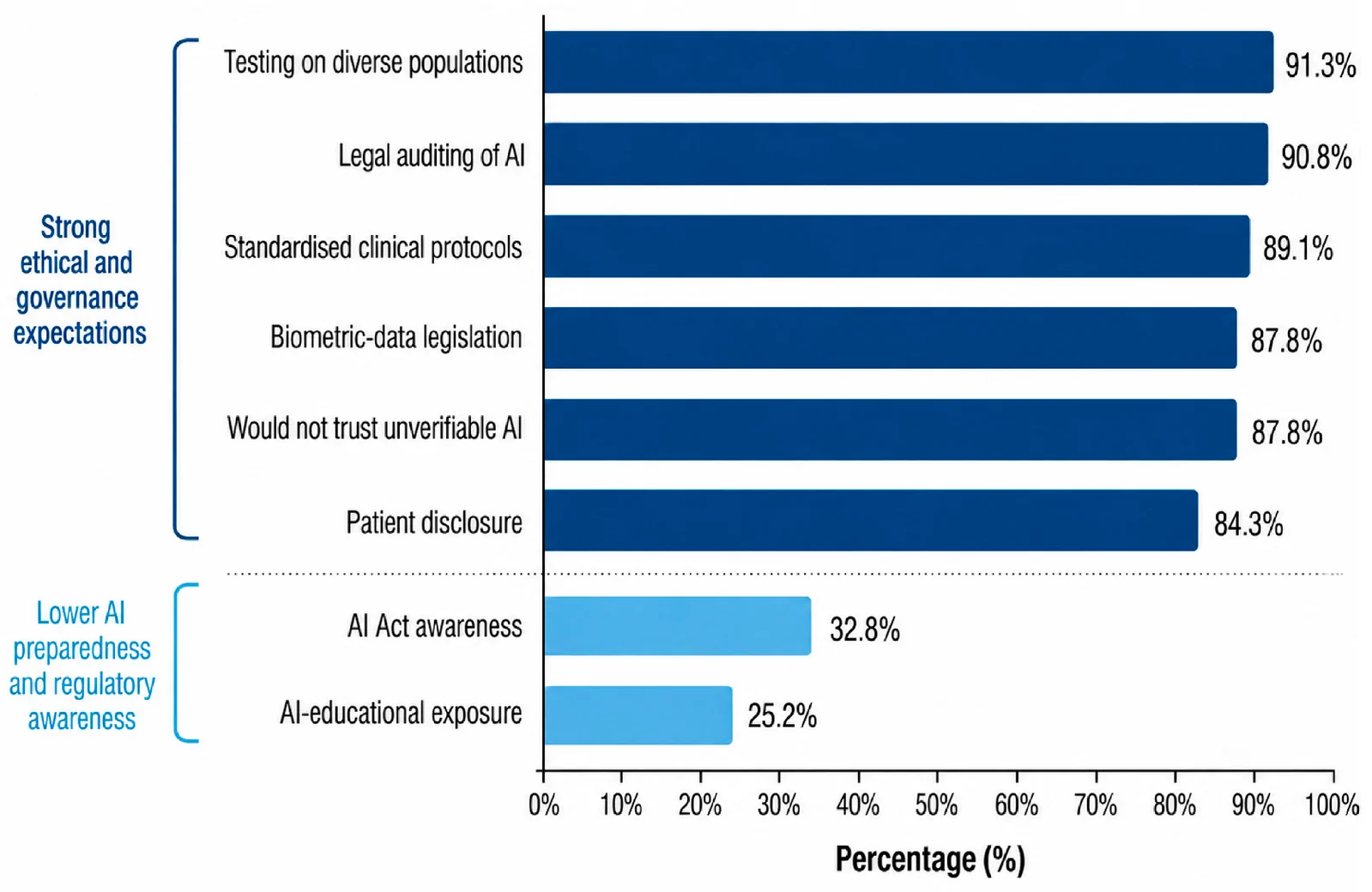
Key Ethical and Governance Findings. Percentages indicate the proportion of respondents endorsing each item or reporting the stated characteristic.

No between-group comparison within the transparency, ethics, and patient-protection domain remained statistically significant after FDR correction, indicating broad cross-stakeholder convergence on the principal requirements for data protection, explainability, patient information, fairness, and regulatory safeguards. The contrast between strong support for ethical and governance safeguards and comparatively limited AI-related educational exposure and regulatory awareness is summarized in Figure 4.

#### 3.2.4. Clinical and Public-Sector Implementation of AI

Participants identified several potential applications for AI in dental services (Q28). Patient-record completion was selected most frequently (74.9%), followed by prosthetic design (67.5%), clinical management (61.5%), patient monitoring (56.9%), and treatment planning (51.9%). Diagnostic support (43.5%), lesion interpretation (42.4%), prognostic assessment (36.0%), and histopathological analysis (30.7%) were selected less frequently. Participants selected a mean of 4.67 potential applications.

Support for selected public-sector applications was high despite concerns regarding implementation. AI-assisted patient triage in public dental services (Q38) was supported by 76.3% of respondents, and 74.8% supported dedicated funding for digitalization and AI through the Romanian National Health Insurance House (CNAS) (Q41). Nevertheless, 86.7% perceived barriers to public-sector implementation (Q40), while respondents rated the realism of implementing AI in public dental clinics within the next five years only moderately (Q39; median, 5; IQR, 3–8), corresponding to the construct referred to analytically as perceived feasibility.

With respect to professional communication and marketing, the median reported frequency of AI use in dental-service promotion was 5 (IQR, 3–8; Q46). Approximately half of respondents (52.4%) supported restricting the use of the term “artificial intelligence” in dental marketing unless AI was demonstrably used in clinical practice (Q47). Legislation (90.2%), standardized clinical protocols (66.4%), and professional guidelines (63.6%) were the measures most frequently selected for preventing misleading AI-related claims (Q48).

The reported frequency of AI use in dental-service promotion was the only outcome in this domain showing a significant adjusted between-group difference (Q46; H(3) = 14.483, *p* = 0.002, FDR-adjusted *p* = 0.044, ε^2^ = 0.041). Dental professionals reported a higher frequency than healthcare stakeholders (median, 6 [IQR, 4–8] vs. 3 [IQR, 1.5–7]), and this difference remained significant following Dunn’s post hoc test with Holm correction (adjusted *p* = 0.003). No other pairwise Q46 comparison remained significant after adjustment.

Unadjusted differences for patient monitoring as a potential application (Q28; *p* = 0.044) and legislation as a measure against misleading AI-related marketing (Q48; *p* = 0.016) did not remain significant after FDR correction. No other comparison within the clinical and public-sector implementation domain remained significant after correction for multiple testing.

### 3.3. Factors Associated with the Perceived Feasibility of Public-Sector AI Integration

Separate exploratory univariable proportional-odds ordinal logistic regression models were used to examine factors associated with the perceived feasibility of implementing AI in public dental clinics within the next five years (Q39; 1–10 scale). Higher exposure to AI-related professional guidelines (Q26) was associated with greater perceived feasibility (OR = 1.18; 95% CI, 1.08–1.30; *p* < 0.001). Similarly, greater self-reported knowledge and application of GDPR requirements in current dental practice (Q19) was associated with higher perceived feasibility (OR = 1.17; 95% CI, 1.08–1.27; *p* < 0.001) (Table 4).

Because the odds ratios were estimated per one-point increase on the respective 1–10 scales, each one-point increase in exposure to AI-related professional guidelines was associated with approximately 18% higher odds of reporting a higher feasibility category, whereas each one-point increase in perceived GDPR knowledge and implementation was associated with approximately 17% higher odds. The models explained a modest proportion of variation in perceived feasibility (Nagelkerke pseudo-R^2^ = 0.044 and 0.051, respectively), consistent with the exploratory nature of the analysis. These findings represent cross-sectional associations and should not be interpreted as evidence of causal effects. Given the modest explanatory capacity of the models, these associations should be regarded as hypothesis-generating signals that warrant further evaluation in multivariable and longitudinal studies.

### 3.4. Exploratory Psychometric Evaluation

Exploratory psychometric analyses were conducted on 28 questionnaire items considered suitable for analysis as ordinal or binary indicators. Sampling adequacy was acceptable (Kaiser–Meyer–Olkin measure = 0.695), and Bartlett’s test of sphericity was statistically significant (χ^2^(378) = 1117.83, *p* < 0.001), supporting the factorability of the item set. Parallel analysis supported retention of four factors.

The four-factor solution was interpretable as representing: (1) governance and accountability safeguards; (2) AI literacy and regulatory awareness; (3) public-sector implementation attitudes, equity, and perceived barriers; and (4) clinical validation, explainability, and professional preparedness. Factor-specific internal consistency estimates are presented in Table 5. Reliability was highest for the governance and accountability factor (α = 0.878; ω = 0.880) and the AI literacy and regulatory awareness factor (α = 0.844; ω = 0.854). The validation, explainability, and professional preparedness factor showed acceptable internal consistency (α = 0.703; ω = 0.720), whereas the implementation, equity, and perceived-barriers factor showed comparatively lower, borderline internal consistency (α = 0.693; ω = 0.696). Overall, factor-specific estimates ranged from α = 0.693 to 0.878 and from ω = 0.696 to 0.880.

Preliminary confirmatory factor analysis of the four-factor solution yielded χ^2^(246) = 380.146, χ^2^/df = 1.55, CFI = 0.885, TLI = 0.871, RMSEA = 0.045, and SRMR = 0.076. The RMSEA, SRMR, and χ^2^/df indicated generally acceptable absolute and residual fit, whereas the CFI and TLI remained below commonly used thresholds for incremental fit. Accordingly, the four-factor structure should be regarded as preliminary rather than independently validated.

Because the exploratory and confirmatory factor analyses were conducted using the same analytical sample, the CFA does not constitute an independent validation of the factor structure. The factor labels should therefore be regarded as descriptive interpretations of the empirical item groupings rather than as established questionnaire subscales. External validation in an independent sample, preferably with prespecified factor structure and assessment of measurement invariance across stakeholder groups, is warranted. Additional methodological and diagnostic details of the exploratory psychometric analysis, including item-selection criteria, the correlation and estimation methods, factor composition, internal-consistency estimates, preliminary CFA fit indices, and inter-factor correlations, are provided in Supplementary Table S7.

### 3.5. Sensitivity Analyses

Sensitivity analyses examining the influence of the heterogeneous healthcare stakeholder group, comparisons involving the smaller forensic physician subgroup, and the inclusion of participants with item-level missing data did not materially alter the overall pattern of the principal statistically significant findings. These analyses supported the robustness of the primary conclusions while reinforcing the need for cautious interpretation of estimates involving the smaller forensic physician subgroup. Detailed sensitivity analyses are provided in Supplementary Table S8.

## 4. Discussion

This multidisciplinary study examined how dental, legal, forensic, and healthcare stakeholders in Romania perceive the conditions required for responsible integration of AI into dental care. Four findings are particularly relevant. First, respondents stated that trust in AI was strongly associated with expectations regarding explainability, traceability, patient information, and continuing human oversight. Second, respondents continued to place primary responsibility on the clinician while simultaneously recognizing responsibilities extending to developers and healthcare institutions. Third, strong agreement regarding governance safeguards coexisted with limited AI-educational exposure and regulatory awareness. Finally, respondents recognized the potential value of AI in public dental services but remained cautious regarding implementation feasibility, highlighting perceived concerns related to institutional readiness, financing, professional guidance, and data-governance arrangements. Overall, the findings were characterized by broad multidisciplinary consensus on core ethical and governance safeguards, more limited profession-specific differences, and a notable contrast between strong normative expectations and comparatively limited self-reported educational and regulatory preparedness. These patterns frame the implications for clinical and institutional governance discussed below.

### 4.1. Foundations of Trustworthy AI Implementation: Explainability, Transparency, and Decision Traceability

One of the clearest findings was that stakeholders did not reduce trustworthy AI to algorithmic performance or explainability alone. Instead, transparency emerged as a broader requirement encompassing the intelligibility of AI recommendations, disclosure to patients, traceability of AI-assisted decisions, and mechanisms for institutional oversight. The importance of AI providing clear explanations received a median score of 10 (IQR, 8–10), and 87.8% of respondents indicated that they would not trust an AI recommendation if its underlying reasoning could not be verified. Importantly, these expectations did not differ significantly across stakeholder groups after correction for multiple comparisons, indicating that explainability is a broadly shared expectation linked to trust in clinical AI across professional groups.

This finding is consistent with the transition in the explainable-AI literature from purely technical interpretability towards clinically meaningful explainability [27]. In healthcare, an explanation has practical value only if it enables clinicians to interrogate an output, relate it to the clinical context, identify potential errors or limitations, and decide whether reliance on the recommendation is justified [9,10,12]. From this perspective, the findings indicate strong support for preserving professional autonomy through clinicians’ continued ability to independently evaluate AI recommendations. However, the present survey assessed stated expectations regarding human oversight and independent professional judgement; it did not examine whether such autonomy is maintained during actual interaction with AI systems or whether clinicians are resistant to automation bias.

Transparency was also understood as extending beyond the clinician–AI interface. A legal obligation to inform patients when AI contributes to therapeutic decision-making was supported by 84.3% of respondents, while 73.1% supported clear identification of automated AI systems used in patient communication. Respondents additionally prioritized clarification of responsibility, patient information, and documentation of training-data sources as components of decision traceability. Together, these findings suggest that transparency was viewed as serving several interconnected purposes: enabling professional scrutiny, preserving patient autonomy, documenting the clinical decision pathway, and facilitating retrospective assessment when an adverse outcome occurs.

The strength of support for governance mechanisms further reinforces this interpretation. Legal auditing of AI algorithms was supported by 90.8% of respondents, while establishment of an ethical oversight body received a median importance score of 10. Explainability therefore appears to have been regarded as necessary but insufficient: stakeholders also expected AI systems to remain auditable and subject to continuing organizational and professional oversight throughout their clinical use.

Previous studies in dentistry have frequently considered explainability primarily in relation to clinician confidence and interpretation of algorithmic outputs [12,18,24]. The present multidisciplinary findings extend this perspective by positioning explainability within a broader governance ecosystem, in which patient disclosure, traceability, auditing, professional judgement, and institutional oversight operate as complementary safeguards.

### 4.2. Professional Responsibility and Perceptions of Accountability in AI-Assisted Dentistry

Responsibility for an incorrect AI-assisted diagnosis was attributed most frequently to the clinician (77.4%), followed by the AI developer (49.5%) and healthcare institution (28.3%). This pattern is important because it suggests that respondents did not perceive AI as transferring clinical responsibility away from the professional using the system. Instead, AI was predominantly viewed as a decision-support technology operating within, rather than replacing, professional judgement.

At the same time, respondents frequently attributed responsibility to more than one actor, indicating that responsibility was perceived as extending across the clinical, technological, and organizational environment in which AI is deployed. This interpretation should be distinguished carefully from legal liability. The present survey assessed stakeholders’ perceptions of responsibility; it did not determine how liability would or should be allocated under civil, professional, or product-liability law. Nevertheless, the findings are compatible with emerging models in which adverse AI-assisted outcomes may arise through interconnected contributions involving system design, validation, procurement, deployment, oversight, and clinical use [14,15,16].

The most notable interprofessional difference concerned institutional responsibility. Healthcare institutions were identified as responsible more frequently by legal professionals (45.7%) and healthcare stakeholders (37.2%) than by dental professionals (20.1%) and forensic physicians (9.5%). The difference remained statistically significant after multiplicity correction. Although the small forensic physician subgroup requires cautious interpretation, the overall pattern may indicate that legal and organizational stakeholders attribute greater importance to institutional duties surrounding system selection, procurement, implementation, supervision, and risk management.

This distinction is relevant because the safety of clinical AI cannot depend exclusively on the clinician at the point of care. Individual professionals generally do not control the provenance of training data, software updates, institutional procurement criteria, cybersecurity arrangements, integration with electronic records, or post-deployment monitoring [24,28]. Responsibility for trustworthy AI implementation therefore necessarily extends beyond individual clinical judgement, even when the clinician retains responsibility for the final clinical decision.

Legal uncertainty was also perceived as a potential implementation barrier. Specific legislation addressing AI-related malpractice was supported by 74.5% of respondents, while 74.0% indicated that they would refrain from using AI if responsibility for errors remained unclear. These responses should not be interpreted as evidence that legal clarification would necessarily increase actual adoption; however, they indicate that uncertainty surrounding responsibility may influence professionals’ willingness to rely on AI-assisted systems.

The strongly preventive orientation of respondents is also noteworthy. Clinical testing, professional validation, standardized protocols, and auditing were all highly supported. Furthermore, traceability and certification garnered substantial endorsement.

Collectively, these findings indicate a preference for governance mechanisms designed to reduce the probability of AI-related harm before questions of retrospective liability arise.

### 4.3. AI Literacy and Professional Preparedness: Normative Consensus Despite Uneven Readiness

AI-related preparedness was substantially less developed than support for AI governance. Only 47.4% of respondents reported familiarity with AI-based healthcare devices, 56.1% reported basic knowledge of AI principles, and 25.2% had previously participated in AI-related courses or conferences. Familiarity with AI testing standards was low (median, 2; IQR, 1–5), as was exposure to professional AI guidelines (median, 1; IQR, 1–3). Q14 (familiarity with AI testing standards) was intended to capture stakeholders’ awareness of the validation and governance context surrounding clinical AI, rather than technical competence in AI testing. These indicators reflect respondents’ self-reported familiarity, knowledge, and educational or regulatory exposure and should not be interpreted as objective measures of AI competence or legal knowledge. Conversely, broad agreement was observed for the inclusion of AI topics in undergraduate education (median, 8; IQR, 5–10), highlighting a clear perceived need for early academic exposure to these technologies. The observed support for curricular integration is consistent with recent evidence from dental education, which emphasizes structured AI education, faculty development, and ethical guidance as important components of sustainable curricular implementation [29].

The coexistence of limited self-reported preparedness and strong governance expectations is one of the more important findings of the study. Despite relatively limited reported familiarity with AI-related knowledge and guidance, respondents strongly endorsed diverse-population testing (91.3%), legal auditing (90.8%), standardized clinical protocols (89.1%), biometric-data safeguards (87.8%), patient disclosure (84.3%), and international certification standards (89.2%). This pattern suggests a distinction between self-reported AI familiarity and normative expectations: stakeholders may report limited knowledge of specific AI systems or regulatory frameworks while still recognizing the conditions under which their use would be considered professionally and ethically acceptable. Direct comparison with previous studies is limited, as the existing dental literature has predominantly examined AI knowledge, attitudes, or educational readiness among dental students, educators, or practitioners, whereas the present study considered clinical, legal, forensic, and healthcare stakeholders within a common framework [30,31].

This distinction is particularly relevant in the European regulatory context. Article 4 of the EU AI Act requires providers and deployers to take measures to support the development of AI literacy among their staff and other persons dealing with the operation and use of AI systems on their behalf, taking into account their technical knowledge, experience, education, training, and context of use. AI literacy obligations under Article 4 have applied since 2 February 2025 [25], while the current wording of Article 4 has been in force since 27 July 2026, following its amendment by Regulation (EU) 2026/1744 [32]. The present findings should not be interpreted as demonstrating compliance with that requirement. Indeed, only 32.8% of participants reported awareness of the AI Act, highlighting a substantial gap between the emerging regulatory environment and respondents’ self-reported awareness of it.

Professional certification before AI use revealed a notable interprofessional difference. Certification was supported by 63.6% overall but less frequently among dental professionals (52.3%) than among legal professionals (78.9%), forensic physicians (76.2%), and healthcare stakeholders (72.1%). This should not necessarily be interpreted as opposition among dental professionals to competence assurance. The high support observed for clinical validation, protocols, system certification, and auditing suggests that respondents may distinguish between governance of the technology and mandatory credentialing of the individual user. Because the survey did not explore the reasons underlying these responses, this interpretation remains tentative.

Educational strategies should therefore extend beyond instruction in software operation. Training for dental professionals should encompass appropriate indications and limitations, interpretation of outputs, recognition of algorithmic bias, data protection, explainability, human oversight, documentation, and responsibility [32]. In this sense, AI literacy should be conceptualized as part of professional competence and clinical judgement rather than as purely technological proficiency.

### 4.4. Fairness, Patient Protection, and Healthcare Justice

The findings also demonstrate that stakeholders considered fairness and patient protection integral to responsible AI implementation. More than half of respondents (61.2%) believed that AI could negatively affect equity in dental care, while 91.3% supported testing AI systems on diverse patient populations before clinical authorization. The contrast between these findings is informative: respondents recognized AI as a potential source of inequity while simultaneously identifying representativeness and pre-implementation testing as important safeguards.

At least two dimensions of equity are relevant. The first concerns representational fairness. AI systems trained or validated on populations that differ systematically from those in which they are deployed may perform unevenly across demographic, clinical, or socioeconomic groups [32]. The strong endorsement of diverse-population testing suggests awareness that acceptable aggregate performance does not necessarily imply comparable performance across all patient groups. This concern is particularly relevant in light of recent evidence from dental imaging AI, where demographic subgroup analyses and equity-related reporting have been found to remain limited, despite generally favourable technical performance [33].

The second concerns distributive fairness. Respondents supported AI-assisted triage in public dental services (76.3%) and dedicated public funding for digitalization and AI (74.8%), yet 86.7% perceived implementation barriers and rated five-year public-sector feasibility only moderately. If advanced AI systems are adopted more rapidly in well-resourced private settings than in public dental services, access to their potential benefits could itself become uneven. Consequently, healthcare justice involves not only whether an algorithm performs fairly but also who can access the clinical environments in which such technologies are available.

Patient protection was similarly evident in attitudes towards data governance. Specific legislation for AI processing of biometric data was supported by 87.8%, while most respondents considered that AI access to electronic health records should require prior patient consent. These findings reinforce the broader interpretation that trustworthy AI was not conceptualized solely in terms of diagnostic performance but also in terms of information rights, data protection, fairness, and meaningful human oversight [32].

### 4.5. Institutional Conditions for Public-Sector AI Implementation: Governance, Financing, and Feasibility

The results indicate that respondents distinguished between the perceived clinical potential of AI and the perceived readiness of the public healthcare system to implement it. Support for AI-assisted triage (76.3%) and dedicated public financing (74.8%) coexisted with widespread recognition of implementation barriers (86.7%) and only moderate perceived feasibility of implementation within the next five years (median, 5; IQR, 3–8). This pattern appears more consistent with a gap in perceived implementation readiness than with rejection of AI itself.

Such a distinction is important because successful adoption depends on more than technology availability. Institutional infrastructure, procurement processes, interoperability, workflow redesign, workforce competence, cybersecurity, data governance, monitoring procedures, and organizational culture all influence whether AI can progress from experimental availability to routine clinical use [28]. The present results therefore support understanding AI implementation as an organizational transformation, not simply the acquisition of new software.

Respondents also distributed implementation responsibilities across several institutional actors. The Ministry of Health was most frequently identified as the principal coordinating institution, while the Romanian College of Dentists, professional societies, and universities were assigned complementary roles in regulation, guideline development, professional oversight, and workforce preparation. This pattern is consistent with a multilayered governance model in which public authorities, professional organizations, healthcare institutions, and academic bodies perform distinct but interdependent functions [25].

The exploratory ordinal regression provides further insight. Greater exposure to AI-related professional guidelines was associated with higher perceived feasibility of AI implementation in public dental services (OR = 1.18; 95% CI, 1.08–1.30; *p* < 0.001). More favourable perceptions of GDPR knowledge and implementation were similarly associated with greater perceived feasibility (OR = 1.17; 95% CI, 1.08–1.27; *p* < 0.001). The corresponding Nagelkerke pseudo-R^2^ values were modest (0.044 and 0.051), indicating that these variables explain only a limited proportion of variation in perceived feasibility. The results should therefore be interpreted as exploratory associations rather than as a predictive model of institutional readiness.

Nevertheless, the direction of these associations is informative. Expectations regarding implementation appear to be related not only to technological familiarity but also to the visibility of professional guidance and confidence in established data-governance arrangements. This supports the view that implementation readiness is partly institutional and regulatory in character rather than exclusively technological.

The EU AI Act provides a useful regulatory comparator for this interpretation. Article 26 [25] establishes obligations for deployers of AI systems classified as high risk, including appropriate technical and organizational measures, human oversight, monitoring, and use in accordance with system instructions. However, the relevance and timing of these obligations depend on the classification of the particular AI system and the applicable transitional framework; they should therefore not be presented as evidence that the dental institutions examined here are presently subject to, prepared for, or compliant with all high-risk-system requirements.

Taken together, these findings indicate that the principal challenge perceived by respondents was not simply whether AI could be useful, but whether healthcare organizations possess the governance structures, financial resources, professional expertise, and infrastructure necessary to deploy it safely and sustainably.

### 4.6. Study Contribution and Practical Implications

The principal contribution of this study lies in examining AI-assisted dentistry simultaneously from clinical, legal, forensic, organizational, and patient-related perspectives. Several previous dental surveys have focused primarily on clinicians’ or students’ knowledge, attitudes, or willingness to adopt AI [18,20,21,22]. The multidisciplinary design used here provides a broader view of the governance environment surrounding clinical AI.

Three practical implications emerge. First, implementation frameworks should preserve clinicians’ ability to critically evaluate AI outputs and exercise independent professional judgement. Second, institutions require explicit procedures spanning procurement, validation, implementation, monitoring, documentation, and incident management, reflecting stakeholders’ perception that responsibility extends beyond the individual clinician. Third, professional education and institutional governance should develop in parallel, combining AI literacy with clear clinical guidance, data safeguards, and organizational oversight.

The involvement of legal professionals was particularly informative because it revealed greater attribution of responsibility to healthcare institutions than was observed among dental professionals. This illustrates the value of multidisciplinary assessment: issues that appear primarily clinical from one professional perspective may be understood as organizational or governance responsibilities from another.

Finally, the broad agreement observed across stakeholder groups should not be overlooked. Many of the most strongly endorsed ethical safeguards—including explainability, diverse-population testing, auditing, patient information, and clinical protocols—showed no significant interprofessional differences after correction for multiple comparisons. The main finding is therefore not simply that stakeholder groups differed, but that substantial multidisciplinary consensus already exists regarding several core conditions for trustworthy AI-assisted dental care.

### 4.7. Strengths and Limitations

Several strengths should be acknowledged. The study included stakeholders from clinical, legal, forensic, institutional, and patient-related backgrounds and applied a common questionnaire across groups, allowing direct comparison of perspectives that are frequently studied separately. Statistical analyses incorporated effect-size estimation, correction for multiple testing, post hoc comparisons, sensitivity analyses, and exploratory psychometric assessment. The study also addressed AI implementation beyond technological acceptance by incorporating explainability, responsibility, patient protection, fairness, regulation, professional preparedness, and public-sector feasibility.

These strengths should be interpreted alongside several limitations. First, the cross-sectional design captures perceptions at a single point in time and precludes causal inference or assessment of temporal changes in these perceptions. This limitation is particularly relevant to the observed associations between exposure to professional guidelines, perceived GDPR implementation, and perceived feasibility of public-sector AI integration.

Second, the study used purposive non-probability sampling and should not be considered nationally representative. Given the exclusively Romanian setting, caution is warranted when extrapolating the findings beyond this national context. Participants were predominantly urban, and approximately two-thirds were located in Bucharest–Ilfov. Professionals with a greater interest in AI, digital dentistry, ethics, or regulation may also have been more likely to participate. Moreover, because invitations sent to institutions could be redistributed further, the total number of individuals who received the survey was unknown, and a conventional response rate could not be calculated.

Third, stakeholder groups were unequal in size. In particular, the forensic physician subgroup was small (n = 21), although this should be considered in the context of the limited size of the forensic medicine workforce in Romania, which comprised only 278 physicians nationally in 2025 [34]. This subgroup reduces the precision of estimates and produces sparse cells for some categorical comparisons. Findings involving this subgroup should therefore be regarded as exploratory. The healthcare stakeholder category was also heterogeneous, combining institutional representatives, patient representatives, and patients; although sensitivity analyses did not materially alter the principal findings, this heterogeneity limits interpretation of that group as a single homogeneous constituency. Direct patient representation was limited, and the findings may therefore not fully capture patient-specific perspectives on AI implementation in dental care.

Fourth, AI literacy, current AI use, implementation readiness, responsibility, and regulatory knowledge were self-reported. The study therefore assesses perceived knowledge, attitudes, and expectations rather than objectively measured AI competence, actual clinical behaviour, institutional readiness, regulatory compliance, or legal liability. Similarly, several broad constructs were represented by single items or relatively brief item sets, and the absence of qualitative interviews limited exploration of the reasons underlying particular responses and interprofessional differences.

Item-level non-response was also substantially higher for the item addressing current professional applications of AI (Q13), which was completed by 228 of 287 participants (79.4%; 59 missing, 20.6%)—by a wide margin the highest non-response of any item in the questionnaire. Critically, this non-response was strongly patterned by professional group (χ^2^(3) = 33.247, *p* < 0.001): the proportion of participants who left the item blank ranged from 9.5% among forensic physicians and 10.6% among dental professionals to 23.3% among healthcare stakeholders and 43.1% among legal professionals. This gradient is consistent with differential applicability of the item, which asks about AI applications in the respondent’s own clinical activity: legal professionals, who do not deliver clinical care, would frequently have no applicable answer, whereas clinically active respondents almost always did. The between-group differences observed for Q13 must therefore be read as comparisons among those stakeholders who reported on current clinical applications, rather than among the four groups in full, and they may reflect differential applicability of the item in addition to differing patterns of AI use. This also reinforces the caution attached to the broader Group 1–versus–Group 2 contrasts on AI familiarity and preparedness, since legal professionals differ from dental professionals not only in their responses but in the very applicability of clinically framed questions. Approaches better suited to non-clinical stakeholders—for example, an explicit “not applicable to my role” response option, or role-tailored item routing—would strengthen future multi-stakeholder instruments of this kind.

Fifth, although multiplicity correction reduced the likelihood of false-positive findings, statistical non-significance should not be interpreted as evidence that stakeholder groups are equivalent. Conversely, statistically significant differences with modest effect sizes should not be overinterpreted as large practical differences.

Finally, the questionnaire was purpose-developed for this study. Exploratory factor analysis supported a four-factor structure, but the preliminary CFA showed mixed fit, with acceptable absolute fit indices and CFI/TLI values below conventional incremental-fit thresholds. One factor also demonstrated comparatively borderline internal consistency. Because EFA and CFA were conducted in the same sample, the structure cannot be considered independently validated. External validation in a larger and more balanced sample is required before the identified factors can be treated as established questionnaire subscales.

In addition, the questionnaire predominantly assessed hypothetical and normative attitudes toward AI in general and did not evaluate participants’ responses to a specific clinical AI system in a real or simulated decision-making context. Accordingly, the findings should not be interpreted as evidence of actual behaviour during AI-assisted clinical decision-making.

Future research should therefore combine broader and more geographically balanced recruitment, including multinational samples, with longitudinal and mixed-methods designs, objectively assess AI competence and institutional readiness, and examine professionals’ behaviour when interacting with specific AI systems, including susceptibility to automation bias and preservation of independent clinical judgement, instead of relying exclusively on hypothetical or self-reported scenarios.

## 5. Conclusions

Across dental professionals, legal professionals, forensic physicians, and healthcare stakeholders included in this Romanian sample, the findings revealed substantial convergence regarding the conditions required for trustworthy AI integration in dentistry. Trust was strongly linked to explainability, verifiable reasoning, patient disclosure, decision traceability, auditing, and institutional oversight. Responsibility for AI-assisted care was attributed primarily to the clinician, but also to developers and healthcare institutions, indicating that stakeholders did not perceive responsibility as confined to the individual practitioner.

At the same time, limited AI-educational exposure, familiarity with testing standards, and regulatory awareness contrasted with strong multidisciplinary support for clinical validation, standardized protocols, diverse-population testing, professional oversight, and legal safeguards. The interprofessional differences that remained after correction for multiple testing were relatively limited and concerned mainly the attribution of institutional responsibility, professional certification before AI use, selected aspects of AI literacy, and AI use in dental-service promotion.

Public-sector findings further indicated a gap between perceived value and perceived implementation readiness: respondents supported AI-assisted triage and dedicated public funding while simultaneously recognizing substantial implementation barriers and only moderate short-term feasibility. Overall, within this sample, responsible AI integration in dentistry emerged as a broader governance challenge likely to involve coordinated progress in professional education, accountability, data protection, financing, infrastructure, and institutional oversight.

These findings provide a multidisciplinary snapshot of expectations surrounding responsible AI implementation in Romanian dentistry and support further development of educational and governance frameworks, while broader and more representative research is required before population-level or policy-effectiveness conclusions can be drawn.

## Figures and Tables

**Table 1:** Prespecified analytical domains, corresponding questionnaire items, and brief item descriptions.

Analytical Domain	Questionnaire Items	No. of Items
AI awareness, literacy, and professional education	Q10, Q11, Q12, Q13, Q14, Q26, Q30, Q31, Q32, Q34	10
Governance, regulation, and professional accountability	Q15, Q16, Q17, Q22, Q23, Q24, Q25, Q27, Q29, Q33, Q37, Q44, Q51, Q52, Q53	15
Transparency, ethics, and patient protection	Q18, Q19, Q20, Q21, Q35, Q36, Q42, Q43, Q45, Q49, Q50	11
Clinical and public-sector implementation	Q28, Q38, Q39, Q40, Q41, Q46, Q47, Q48	8

**Table 2:** Descriptive statistics for all 1–10 ordinal outcomes related to AI preparedness, governance, transparency, and implementation.

Item	Construct	Valid n	Missing n	Median	IQR (P25–P75)
Q14	Familiarity with AI testing standards	287	0	2	1–5
Q15	Importance of clinical testing before AI implementation	287	0	10	9–10
Q17	Importance of professional involvement in AI validation	287	0	10	8–10
Q18	Perceived risk to patient-data confidentiality	286	1	5	2–7
Q19	Knowledge/application of GDPR in current practice	285	2	6	5–8
Q26	Exposure to AI-related professional guidelines	285	2	1	1–3
Q31	Importance of introducing AI into undergraduate curricula	287	0	8	5–10
Q37	Importance of an ethical oversight body for healthcare AI	287	0	10	7–10
Q39	Perceived feasibility of AI implementation in public dental clinics within five years	287	0	5	3–8
Q42	Importance of clear explanations for AI decisions	287	0	10	8–10
Q46	Frequency of AI use in dental-service promotion	284	3	5	3–8
Q50	Importance of aligning Romanian legislation with the EU AI Act	287	0	10	8–10

Responses were recorded on 1–10 scales using item-specific anchors. Data are presented as median and interquartile range (IQR) because these items were treated as ordinal variables in inferential analyses. Missing values were handled using available-case analysis. GDPR = General Data Protection Regulation; AI = artificial intelligence. Means and standard deviations are provided in Supplementary Table S6.

**Table 3:** Between-group differences that remained significant after correction for multiple comparisons.

Item/Outcome	Valid n	Global Test	Global *p*	Global *p* (FDR-Adjusted)	Effect Size	Significant Adjusted Pairwise Comparisons
Q10 Familiarity with AI-based healthcare devices	287	χ^2^(3) = 20.093	<0.001	<0.001	V = 0.265	G1 vs. G2, *p*FDR < 0.001
Q11 Basic knowledge of AI principles	287	χ^2^(3) = 38.018	<0.001	<0.001	V = 0.364	G1 vs. G2 < 0.001; G1 vs. G4 = 0.021; G2 vs. G3 = 0.021; G2 vs. G4 = 0.042
Q13 Clinical management	228	χ^2^(3) = 18.719	<0.001	0.001	V = 0.287	G1 vs. G3 = 0.001; G1 vs. G4 = 0.048
Q13 Treatment planning	228	χ^2^(3) = 19.105	<0.001	0.001	V = 0.289	G1 vs. G2 = 0.020; G1 vs. G3 = 0.011; G1 vs. G4 = 0.020
Q13 Other applications	228	χ^2^(3) = 11.162	0.011	0.029	V = 0.221	G1 vs. G2 = 0.022
Q13 No current AI use	228	χ^2^(3) = 15.256	0.002	0.005	V = 0.259	G1 vs. G2 = 0.006; G1 vs. G3 = 0.040
Q14 Familiarity with AI testing standards	287	H(3) = 23.830	<0.001	<0.001	ε^2^ = 0.074	G1 vs. G2 < 0.001; G1 vs. G3 = 0.014
Q15 Importance of clinical testing before AI implementation	287	H(3) = 13.159	0.004	0.047	ε^2^ = 0.036	G2 vs. G3 = 0.025
Q26 Exposure to AI-related professional guidelines	285	H(3) = 32.971	<0.001	<0.001	ε^2^ = 0.107	G1 vs. G2 < 0.001; G1 vs. G3 = 0.002; G1 vs. G4 = 0.043
Q30 Participation in AI-related courses/conferences	287	χ^2^(3) = 49.690	<0.001	<0.001	V = 0.417	G1 vs. G2 < 0.001; G1 vs. G3 = 0.002; G1 vs. G4 = 0.004; G2 vs. G4 = 0.007
Q22_3 Institution selected as responsible	283	χ^2^(3) = 20.703	<0.001	0.004	V = 0.270	G1 vs. G2 = 0.001; G2 vs. G3 = 0.006
Q33 Professional certification before AI use	286	χ^2^(3) = 18.242	<0.001	0.006	V = 0.253	G1 vs. G2 = 0.001
Q46 Frequency of AI use in dental-service promotion	284	H(3) = 14.483	0.002	0.044	ε^2^ = 0.041	G1 > G4, *p*Holm = 0.003

G1 = dental professionals; G2 = legal professionals; G3 = forensic physicians; G4 = healthcare stakeholders. All outcomes shown remained statistically significant after the prespecified domain-level Benjamini–Hochberg FDR correction. Pairwise binary comparisons were adjusted using the Benjamini–Hochberg procedure; †ordinal pairwise comparisons were performed using Dunn’s test with Holm correction. Complete analyses, including comparisons not retained after correction, are reported in Supplementary Tables S4 and S5. Analyses were conducted using available cases; the valid n is shown for each outcome. Q13 was answered by 228 participants, and its effect sizes are therefore calculated on that denominator.

**Table 4:** Exploratory univariable ordinal logistic regression analyses of associations with perceived feasibility of AI implementation in public dental clinics.

Predictor	n	OR per 1-Point Increase	95% CI	*p*	Model LR χ^2^	LR *p*	Parallel-Lines χ^2^	Parallel-Lines *p*	Nagelkerke *R*^2^
Exposure to AI-related professional guidelines (Q26)	285	1.18	1.08–1.30	<0.001	12.72	<0.001	χ^2^(8) = 7.09	0.527	0.044
Knowledge and application of GDPR requirements in current dental practice (Q19)	285	1.17	1.08–1.27	<0.001	14.68	<0.001	χ^2^(8) = 16.53	0.035	0.051

Q39 was the ordinal dependent variable and assessed the perceived feasibility of implementing AI in public dental clinics within the next five years on a 1–10 scale. Q19 and Q26 were also measured on 1–10 scales and were examined in separate unadjusted exploratory proportional-odds models. OR = odds ratio; CI = confidence interval; LR = likelihood-ratio test. An OR > 1 indicates greater odds of reporting a higher perceived-feasibility category for each one-point increase in the predictor. Available-case analysis was used.

**Table 5:** Internal consistency of the four-factor exploratory questionnaire structure.

Factor	Items	No. of Items	Cronbach’s α	McDonald’s ω
Governance and accountability safeguards	Q20, Q23, Q25, Q27, Q33, Q36, Q37, Q44	8	0.878	0.880
AI literacy and regulatory awareness	Q10, Q11, Q14, Q26, Q30, Q34, Q51	7	0.844	0.854
Public-sector implementation attitudes, equity, and perceived barriers	Q18, Q35, Q38, Q39, Q40, Q41, Q43	7	0.693	0.696
Clinical validation, explainability, and professional preparedness	Q15, Q17, Q19, Q31, Q42, Q50	6	0.703	0.720

α = Cronbach’s alpha; ω = McDonald’s omega. Factor labels represent descriptive interpretations of the exploratory factor solution and should not be interpreted as independently validated questionnaire subscales.

## Data Availability

The data supporting the findings of this study are available from the first author or the corresponding author upon reasonable request. The dataset is not being made publicly available at this stage because it forms part of the first author’s PhD thesis, which has not yet been defended.

## Supplementary Materials

The following supporting information can be downloaded at https://www.mdpi.com/article/10.3390/healthcare14183003/s1. Table S1, Completed CROSS checklist; Table S2, Questionnaire (English versions); Table S3, Demographic and professional characteristics of the analytical sample; Table S4, Complete global between-group test results for all questionnaire outcomes, including unadjusted and domain-level FDR-adjusted *p*-values, test statistics, effect sizes, and valid sample sizes; Table S5, Complete adjusted pairwise post hoc comparison results; Table S6, Means and standard deviations for all 1–10 ordinal outcomes; Table S7, Exploratory psychometric evaluation of the questionnaire, including item-selection criteria, analytical approach, factor composition, internal-consistency estimates, preliminary CFA fit indices, and inter-factor correlations; Table S8, Sensitivity-analysis. Refs. [5,12,35,36,37] are cited in Supplementary Materials.

## Abbreviations

The following abbreviations are used in this manuscript:

AI	Artificial intelligence
EU	European Union
GDPR	General Data Protection Regulation
FDI	Fédération Dentaire Internationale (World Dental Federation)
WHO	World Health Organization
CROSS	Consensus-Based Checklist for Reporting of Survey Studies
CNAS	Casa Națională de Asigurări de Sănătate (Romanian National Health Insurance House)
SD	Standard deviation
IQR	Interquartile range
OR	Odds ratio
CI	Confidence interval
LR	Likelihood ratio
FDR	False discovery rate
EFA	Exploratory factor analysis
CFA	Confirmatory factor analysis
CFI	Comparative fit index
TLI	Tucker–Lewis index
RMSEA	Root mean square error of approximation
SRMR	Standardized root mean square residual
SPSS	Statistical Package for the Social Sciences
