# Supplementary material for: Responsible Clinical AI in Dentistry: Trust, Professional Autonomy, and Perceptions of Accountability Across Stakeholders in Romania—A Multidisciplinary Cross-Sectional Survey
Source: Healthcare (Basel). 2026 Sep 14;14(18):3003. doi: 10.3390/healthcare14183003 (PMC13606574; doi:10.3390/healthcare14183003)
Supplement: Supplementary file 1 [file healthcare-14-03003-s001.zip › Supplementary Material S2._English_language_version_questionnaire.pdf]

healthcare

# QUESTIONNAIRE ON THE USE OF ARTIFICIAL INTELLIGENCE IN DENTAL PRACTICE

*Table S2:* "Responsible Clinical AI in Dentistry: Trust, Professional Autonomy, and Perceptions of Accountability Across Stakeholders in Romania — A Multidisciplinary Cross-Sectional Survey"

| Nr. | Item | Answer | Supporting framework/source(s) |
|---|---|---|---|
| Q1 | E-mail address:<br>(Please fill in) | | Study protocol and data-management procedure |
| Q2 | I agree to participate in this study: | YES<br>NO | Study protocol; Ethics Committee-approved participant information and consent procedure |
| Q3 | Do you consent to the anonymous processing of the data you provide for research purposes? | YES<br>NO | GDPR; study data-management procedure |
| Q4 | Age:<br>(Please fill in) | | Standard demographic variable |
| Q5 | Gender | 1. Male<br>2. Female<br>3. Prefer not to say | Standard demographic variable |
| Q6 | Professional category/Speciality: | o Dentist (specialty: general dentistry / prosthodontics / endodontics / orthodontics / pediatric dentistry / periodontology / DTDO / dentoalveolar surgery / maxillofacial surgery)<br>o Dental technician<br>o Dental equipment and materials manufacturer<br>o Forensic physicians<br>o Judge<br>o Prosecutor<br>o Lawyer<br>o Legal advisor<br>o Patient<br>o Patient representative<br>o Ministry of Health (representative)<br>o National Public Health Authority (representative)<br>o County Public Health Office (representative)<br>o National Institute of Public Health (representative)<br>o Student<br>o Other | Study sampling framework |

healthcare

## QUESTIONNAIRE ON THE USE OF ARTIFICIAL INTELLIGENCE IN DENTAL PRACTICE

| | | | |
|---|---|---|---|
| Q7 | Are you a university faculty member? (Select one response only) | YES<br>NO | Study sampling framework |
| Q8 | Region: | Bucharest and Ilfov<br>Centre<br>North<br>South<br>East<br>West<br>Nordeast<br>Nordwest<br>Southeast<br>Southwest | Romanian development-region classification used by the study |
| Q9 | Environment: | Urban<br>Rural | Study sampling framework |
| Q10 | Are you familiar with AI-based healthcare devices? (Select one response only) | YES<br>NO | WHO 2021; FDI 2024; AI Act |
| Q11 | Do you have basic knowledge of the principles underlying the application of AI in medicine? (Select one response only) | YES<br>NO | WHO 2021; FDI 2024; AI Act |
| Q12 | What do you think are the advantages of using AI in medicine? (Select all that apply) | o AI can provide large amounts of high-quality, clinically relevant data in real time<br>o AI can assist in decision-making<br>o AI can support the diagnostic process<br>o AI can reduce medical errors<br>o AI can accelerate healthcare processes<br>o AI does not suffer from emotional exhaustion or physical limitations<br>o Other | WHO 2021; FDI 2024; AI Act |
| Q13 | As a practitioner, you use AI for: (Select all that apply) | o Clinical management<br>o Completing patient records<br>o Patient follow-up/monitoring<br>o Diagnosis<br>o Treatment planning<br>o Short- or long-term prognosis<br>o Lesion interpretation<br>o Histopathological analysis<br>o Other<br>• I do not use AI | FDI 2024; WHO 2021; GDPR for patient-record functions |
| Q14 | How familiar are you with current standards for testing AI systems in dental medicine? Please rate from 1 to 10, where: 1 = "not familiar at all", | 1 2 3 4 5 6 7 8 9 10 | AI Act; WHO 2021; FDI 2024 |

| | | | |
|---|---|---|---|
| | and 10 = "very familiar". (Select one response only) | | |
| Q15 | Do you think AI systems should undergo clinical testing similar to that required for medical devices before clinical implementation? Please rate your level of agreement on a scale from 1 to 10, where: 1 = "strongly disagree", and 10 = "strongly agree". (Select one response only) | 1 2 3 4 5 6 7 8 9 10 | AI Act; WHO 2021; FDI 2024; Rokhshad et al. 2023 |
| Q16 | Should mandatory evaluation of AI performance be regulated by national or European legislation? (Select one response only) | 1. European legislation<br>2. National legislation<br>3. Both European and national legislation<br>4. Other | AI Act; WHO 2021; FDI 2024; Rokhshad et al. 2023 |
| Q17 | What level of involvement should dental professionals have in the validation of AI before clinical implementation? Please rate from 1 to 10, where: 1 = "no involvement", and 10 = "full involvement". (Select one response only) | 1 2 3 4 5 6 7 8 9 10 | WHO 2021; FDI 2024; AI Act; Rokhshad et al. 2023 |
| Q18 | To what extent do you think that the use of AI in dentistry endangers patient data confidentiality? Please rate from 1 to 10, where: 1 = "no risk at all", and 10 = "very high risk". (Select one response only) | 1 2 3 4 5 6 7 8 9 10 | GDPR; WHO 2021; FDI 2024; Rokhshad et al. 2023 |
| Q19 | How well do you think GDPR regulations are known and applied in current dental practice? Please rate from 1 to 10, where: 1 = "not at all", and 10 = "very well known and applied". (Select one response only) | 1 2 3 4 5 6 7 8 9 10 | GDPR; WHO 2021; FDI 2024; Rokhshad et al. 2023 |
| Q20 | Is specific legislation needed for the processing of biometric data (e.g., facial images, intraoral scans) by AI? (Select one response only) | YES<br>NO | GDPR; AI Act; WHO 2021; FDI 2024; Rokhshad et al. 2023 |
| Q21 | Would you agree for AI to automatically access data from the patient's electronic health record to suggest treatments? * (Select one response only) | 1. Yes<br>2. Yes, with the patient's prior consent<br>3. No | GDPR; WHO 2021; FDI 2024; AI Act; Rokhshad et al. 2023 |

| | | | |
|---|---|---|---|
| Q22 | Who do you think should be held responsible in the event of an incorrect diagnosis provided by an AI system? (Select all that apply) | o The AI developer<br>o The clinician<br>o The healthcare institution<br>o Other. | WHO 2021; FDI 2024; Rokhshad et al. 2023 |
| Q23 | Is a distinct legal framework needed for AI-assisted malpractice? (Select one response only) | YES<br>NO | WHO 2021; FDI 2024; Rokhshad et al. 2023 |
| Q24 | What measures should be in place the traceability AI-assisted decisions? (Select all that apply) | o Documentation of the data sources used to train the model<br>o Clear allocation of responsibility between developer and physician – who is liable if the AI makes an error?<br>o Archiving decisions and outcomes for each clinical case<br>o Providing patiens with clear information about the use of AI in the decision-making process<br>o Periodic ethical evaluations of how decisions are made and communicated<br>o Other | AI Act; WHO 2021; GDPR; Rokhshad et al. 2023 |
| Q25 | Would you avoid using AI software if legal liability is not clearly regulated? (Select one response only) | YES<br>NO | WHO 2021; FDI 2024; Rokhshad et al. 2023 |
| Q26 | How often have you encountered, in the professional literature, guidelines addressing the use of AI in dentistry?* Please rate from 1 to 10, where: 1 = "never encountered", and 10 = "very frequently encountered". (Select one response only) | 1 2 3 4 5 6 7 8 9 10 | FDI 2024; WHO 2021; AI Act |
| Q27 | Do you consider standardised national protocols for the use of AI in dental care necessary ? (Select one response only) | YES<br>NO | FDI 2024; WHO 2021; AI Act; Rokhshad et al. 2023 |
| Q28 | What dental interventions would be most suitable for AI integration? (Select all that apply) | o Clinical management<br>o Patient record completion<br>o Follow-up / Monitoring<br>o Diagnosis<br>o Treatment planning<br>o Prosthetic design<br>o Short- or long-term prognosis<br>o Lesion interpretation<br>o Histopathological analysis<br>o Other | FDI 2024 |

| | | | |
|---|---|---|---|
| Q29 | Who should be responsible at the national level for developing clinical guidelines for the use of AI in dental treatments? (Select all that apply) | o Professional associations<br>o Ministry of Health<br>o Universities<br>o AI developers<br>o Civil society<br>o Other | FDI 2024; WHO 2021; Rokhshad et al. 2023 |
| Q30 | Have you previously attended any courses or conferences on the use of AI in dental medicine so far? (Select one response only) | YES<br>NO | FDI 2024; AI Act |
| Q31 | How important do you consider the integration of AI into undergraduate dental curricula? Please rate from 1 to 10, where: 1 = "not important at all", and 10 = "very important" (Select one response only) | 1 2 3 4 5 6 7 8 9 10 | FDI 2024; WHO 2021 |
| Q32 | Which type of training do you consider most useful (hands-on workshop, software training, webinar, etc.)? (Select one response only) | 1. Hands-on workshop<br>2. Software training<br>3. Webinar<br>4. Other | FDI 2024; WHO 2021 |
| Q33 | Should the use of AI in dental practice be conditional upon professional certification? (Select one response only) | YES<br>NO | WHO 2021; FDI 2024; Rokhshad et al. 2023 |
| Q34 | Are you familiar with the term "algorithmic bias" (systematic unfairness or sampling bias arising from the data used to train an AI system)? (Select one response only) | YES<br>NO | WHO 2021; FDI 2024; Rokhshad et al. 2023 |
| Q35 | Do you think AI could negatively affect equity in dental care, for example through the underrepresentation of certain patient groups? (Select one response only) | YES<br>NO | WHO 2021; FDI 2024; Rokhshad et al. 2023 |
| Q36 | Should AI systems be required by regulation to be tested on a diverse sample of patients before authorisation? (Select one response only) | YES<br>NO | AI Act; WHO 2021; FDI 2024; Rokhshad et al. 2023 |

| | | | |
|---|---|---|---|
| Q37 | How important do you consider the establishment of an independent ethical oversight body for AI in healthcare? Please rate from 1 to 10, where: 1 = "not important at all", and 10 = "very important". (Select one response only) | 1 2 3 4 5 6 7 8 9 10 | WHO 2021; FDI 2024; Rokhshad et al. 2023 |
| Q38 | Do you think AI could be useful for patient triage in the public dental healthcare system? (Select one response only) | YES<br>NO | WHO 2021; FDI 2024 |
| Q39 | How realistic do you consider the integration of AI into public dental clinics over the next five years? Please rate from 1 to 10, where: 1 = "completely unrealistic", and 10 = "very realistic". (Select one response only) | 1 2 3 4 5 6 7 8 9 10 | WHO 2021; FDI 2024 |
| Q40 | Do you think there would be barriers to the adoption of AI in the public sector related to  resources, training, or infrastructure? (Select one response only) | YES<br>NO | FDI 2024; WHO 2021 |
| Q41 | Should the buget of the Roumanian National Health Insurance House (CNAS) include dedicated funding for digitalisation and AI in dentistry? (Select one response only) | YES<br>NO | FDI 2024 |
| Q42 | How important is it for AI systems  to provide clear explanations for their recommendations or decisions that is, to be explainable (i.e., “explainable” systems)?Please rate from 1 to 10, where: 1 = "not important at all", and 10 = "very important".(Select one response only) | 1 2 3 4 5 6 7 8 9 10 | AI Act; WHO 2021; FDI 2024; Rokhshad et al. 2023 |
| Q43 | Would you trust an AI-generated recommendation if you could not verify the reasoning behind the decision? (Select one response only) | YES<br>NO | WHO 2021; FDI 2024; Rokhshad et al. 2023 |
| Q44 | Do you consider a legal mechanism for auditing AI algorithms used in | YES<br>NO | AI Act; WHO 2021; FDI 2024; Rokhshad et al. 2023 |

| | | | |
|---|---|---|---|
| | dental clinics necessary? (Select one response only) | | |
| Q45 | Should there be a legal obligation to inform the patient if AI has been used in tratament decision-making? (Select one response only) | YES<br>NO<br>I DON'T KNOW | AI Act; WHO 2021; FDI 2024; Rokhshad et al. 2023 |
| Q46 | How frequently do you think AI is currently used to promote dental services? Please rate from 1 to 10, where: 1 = "very rarely", and 10 = "very frequently".(Select one response only) | 1 2 3 4 5 6 7 8 9 10 | AI Act; WHO 2021 |
| Q47 | Should the use of the term "artificial Intelligence" in dental marketing be prohibited unless there is evidence that AI is actually being used? (Select one response only) | YES<br>NO<br>I DON'T KNOW | WHO 2021; FDI 2024; Rokhshad et al. 2023 |
| Q48 | What type of regulation do you consider appropriate to prevent misleading AI-related advertising? (Select all that apply) | o Legislation<br>o Standardized working protocols<br>o Practice guidelines<br>o Other | WHO 2021; FDI 2024 |
| Q49 | Should all AI applications used to communicate with patients be clearly labelled as "automated systems"? (Select one response only) | YES<br>NO<br>I DON'T KNOW | AI Act; WHO 2021; FDI 2024; Rokhshad et al. 2023 |
| Q50 | How important is it for Romanian legislation to be aligned with the European proposals concerning the AI Act? Please rate from 1 to 10, where: 1 = "not important at all", and 10 = "very important". (Select one response only) | 1 2 3 4 5 6 7 8 9 10 | AI Act; WHO 2021; FDI 2024 |
| Q51 | Are you aware of the Artificial Intelligence Act (AI Act) proposed by the European Commission ? (Select one response only) | YES<br>NO | AI Act; WHO 2021; FDI 2024 |
| Q52 | Which Romanian institutions should actively participate in the implementation of these regulations in healthcare? (Select all that apply.) | o Ministry of Health<br>o Ministry of Justice<br>o Developer companies<br>o College of Dentists<br>o Professional societies<br>o Universities<br>o Other | AI Act; WHO 2021; FDI 2024; Rokhshad et al. 2023 |

| | | | |
|---|---|---|---|
| Q53 | Should international standards be adopted for the certification of AI applications in dentistry? (Select one response only) | YES<br>NO<br>I DON'T KNOW | AI Act; WHO 2021; FDI 2024; Rokhshad et al. 2023 |

## References

World Health Organization. Ethics and governance of artificial intelligence for health: WHO guidance. Geneva: World Health Organization; 2021. ISBN 978-92-4-002920-0.

Rokhshad R, Ducret M, Chaurasia A, et al. Ethical considerations on artificial intelligence in dentistry: A framework and checklist. Journal of Dentistry. 2023;135:104593. doi:10.1016/j.jdent.2023.104593.

FDI World Dental Federation. Artificial intelligence in dentistry. Policy statement adopted by the FDI General Assembly, Istanbul, Türkiye, September 2024.

European Parliament and Council of the European Union. Regulation (EU) 2024/1689 laying down harmonised rules on artificial intelligence (Artificial Intelligence Act). Official Journal of the European Union. 2024.

European Parliament and Council of the European Union. Regulation (EU) 2016/679 (General Data Protection Regulation). Official Journal of the European Union. 2016.
