# Supplementary material for: Responsible Clinical AI in Dentistry: Trust, Professional Autonomy, and Perceptions of Accountability Across Stakeholders in Romania—A Multidisciplinary Cross-Sectional Survey
Source: Healthcare (Basel). 2026 Sep 14;14(18):3003. doi: 10.3390/healthcare14183003 (PMC13606574; doi:10.3390/healthcare14183003)
Supplement: Supplementary file 1 [file healthcare-14-03003-s001.zip › Supplementary Material S3.pdf]

Supplementary Material S3

Table S3. Demographic and professional characteristics of the analytical sample (n = 287).

Characteristic	Category / Statistic	n (%) or Summary
**Age, years**	Valid responses	275
	Mean ± SD	43.9 ± 10.2
	Median (IQR)	44 (36–51)
	Range	19–69
**Gender**	Female	166 (58.0)
	Male	120 (42.0)
**Professional group**	Dental professionals	151 (52.6)
	Legal professionals	72 (25.1)
	Forensic physicians	21 (7.3)
	Healthcare stakeholders	43 (15.0)
**Academic staff status**	Yes	118 (41.4)
	No	167 (58.6)
**Geographical region**	Bucharest–Ilfov	189 (66.1)
	Centre	44 (15.4)
	North-West	20 (7.0)
	South-East	11 (3.8)
	South	10 (3.5)
	North-East	7 (2.4)
	West	5 (1.7)
**Area of residence**	Urban	278 (96.9)
	Rural	9 (3.1)

Data are presented as n (%) unless otherwise indicated. Percentages were calculated using the number of valid responses for each variable and may not sum to 100% because of rounding. Missing data were as follows: age, n = 12; gender, n = 1; academic staff status, n = 2; and geographical region, n = 1. There were no missing data for professional group or area of residence.
