# Supplementary material for: Responsible Clinical AI in Dentistry: Trust, Professional Autonomy, and Perceptions of Accountability Across Stakeholders in Romania—A Multidisciplinary Cross-Sectional Survey
Source: Healthcare (Basel). 2026 Sep 14;14(18):3003. doi: 10.3390/healthcare14183003 (PMC13606574; doi:10.3390/healthcare14183003)
Supplement: Supplementary file 1 [file healthcare-14-03003-s001.zip › Supplementary_Materials_S4-S8.pdf]

Supplementary Materials

Responsible Clinical AI in Dentistry: Trust, Professional Autonomy, and Perceptions of Accountability Across Stakeholders in Romania — A Multidisciplinary Cross-Sectional Survey

Table S4. Complete global between-group test results for all questionnaire items

Global comparisons across the four stakeholder groups (G1 = dental professionals, n = 151; G2 = legal professionals, n = 72; G3 = forensic physicians, n = 21; G4 = healthcare stakeholders, n = 43). Categorical outcomes were compared using the Pearson chi-square test and 1–10 ordinal outcomes using the Kruskal–Wallis test. Each response option of a multiple-response item was analysed as a separate binary variable. p (FDR-adjusted) values were obtained with the Benjamini–Hochberg procedure applied within each analytical domain. Effect sizes: V = Cramér's V; ε^2 = epsilon-squared. Available-case analysis; valid n is shown for each outcome.

Domain	Item / outcome	Global test	Global p	p (FDR-adj.)	Effect size	Valid n	Sig. after FDR
AI literacy & education	**Q30 Participation in AI-related courses/conferences**	$\chi^2(3)$ = 49.690	<0.001	**<0.001**	V = 0.417	287	Yes
AI literacy & education	**Q11 Basic knowledge of AI principles**	$\chi^2(3)$ = 38.018	<0.001	**<0.001**	V = 0.364	287	Yes
AI literacy & education	**Q26 Exposure to AI-related professional guidelines**	H(3) = 32.971	<0.001	**<0.001**	ε^2 = 0.107	285	Yes
AI literacy & education	**Q14 Familiarity with AI testing standards**	H(3) = 23.830	<0.001	**<0.001**	ε^2 = 0.074	287	Yes
AI literacy & education	**Q10 Familiarity with AI-based healthcare devices**	$\chi^2(3)$ = 20.093	<0.001	**<0.001**	V = 0.265	287	Yes
AI literacy & education	**Q13 — treatment planning**	$\chi^2(3)$ = 19.105	<0.001	**0.001**	V = 0.289	228	Yes
AI literacy & education	**Q13 — clinical management**	$\chi^2(3)$ = 18.719	<0.001	**0.001**	V = 0.287	228	Yes
AI literacy & education	**Q13 — no current AI use**	$\chi^2(3)$ = 15.256	0.002	**0.005**	V = 0.259	228	Yes
AI literacy & education	**Q13 — other applications**	$\chi^2(3)$ = 11.162	0.011	**0.029**	V = 0.221	228	Yes
AI literacy & education	Q12 — AI can provide large amounts of high-quality clinically relevant data in real time	$\chi^2(3)$ = 8.762	0.033	0.077	V = 0.175	287	No
AI literacy & education	Q13 — lesion interpretation	$\chi^2(3)$ = 8.385	0.039	0.077	V = 0.192	228	No
AI literacy & education	Q13 — histopathological analysis	$\chi^2(3)$ = 8.383	0.039	0.077	V = 0.192	228	No
AI literacy & education	Q13 — patient monitoring	$\chi^2(3)$ = 7.899	0.048	0.089	V = 0.186	228	No
AI literacy & education	Q13 — patient record completion	$\chi^2(3)$ = 7.595	0.055	0.095	V = 0.183	228	No
AI literacy & education	Q13 — diagnostic support	$\chi^2(3)$ = 7.300	0.063	0.101	V = 0.179	228	No
AI literacy & education	Q32 Preferred educational formats	$\chi^2(12)$ = 18.761	0.094	0.142	V = 0.149	283	No
AI literacy & education	Q12 — AI can assist in the diagnostic process	$\chi^2(3)$ = 4.647	0.200	0.282	V = 0.127	287	No
AI literacy & education	Q34 Familiarity with the term algorithmic bias	$\chi^2(3)$ = 3.270	0.352	0.456	V = 0.107	286	No
AI literacy & education	Q12 — AI can reduce medical errors	$\chi^2(3)$ = 3.203	0.361	0.456	V = 0.106	287	No
AI literacy & education	Q13 — short- or long-term prognostic assessment	$\chi^2(3)$ = 2.683	0.443	0.532	V = 0.108	228	No
AI literacy & education	Q12 — AI can assist decision-making	$\chi^2(3)$ = 1.680	0.641	0.733	V = 0.077	287	No
AI literacy & education	Q31 Importance of AI in undergraduate curricula	H(3) = 1.419	0.701	0.764	ε^2 = -0.006	287	No
AI literacy & education	Q12 — AI does not experience emotional exhaustion or physical limitations	$\chi^2(3)$ = 1.287	0.732	0.764	V = 0.067	287	No
AI literacy & education	Q12 — AI can accelerate healthcare processes	$\chi^2(3)$ = 1.139	0.768	0.768	V = 0.063	287	No
Governance & accountability	**Q22 — institution responsible**	$\chi^2(3)$ = 20.703	<0.001	**0.004**	V = 0.270	283	Yes

Governance & accountability	**Q33 Support for professional certification before AI use**	$\chi^2(3) = 18.242$	<0.001	**0.006**	V = 0.253	286	Yes
Governance & accountability	**Q15 Importance of clinical testing before AI implementation**	H(3) = 13.159	0.004	**0.047**	$\varepsilon^2 = 0.036$	287	Yes
Governance & accountability	Q25 Would refrain from AI use if liability unclear	$\chi^2(3) = 11.685$	0.009	0.070	V = 0.202	285	No
Governance & accountability	Q52 — Ministry of Justice	$\chi^2(3) = 9.771$	0.021	0.119	V = 0.185	285	No
Governance & accountability	Q24 — Archiving decisions and outcomes for each clinical case	$\chi^2(3) = 9.654$	0.022	0.119	V = 0.183	287	No
Governance & accountability	Q24 — Documentation of the source of data used to train the model	$\chi^2(3) = 9.250$	0.026	0.119	V = 0.180	287	No
Governance & accountability	Q29 — Universities	$\chi^2(3) = 8.989$	0.029	0.119	V = 0.177	286	No
Governance & accountability	Q29 — Developer companies	$\chi^2(3) = 8.771$	0.032	0.119	V = 0.175	286	No
Governance & accountability	Q22 — other applications	$\chi^2(3) = 7.638$	0.054	0.179	V = 0.164	283	No
Governance & accountability	Q29 — Professional societies	$\chi^2(3) = 6.901$	0.075	0.225	V = 0.155	286	No
Governance & accountability	Q29 — Civil society	$\chi^2(3) = 6.547$	0.088	0.242	V = 0.151	286	No
Governance & accountability	Q29 — Ministry of Health	$\chi^2(3) = 5.544$	0.136	0.327	V = 0.139	286	No
Governance & accountability	Q52 — Developer companies	$\chi^2(3) = 5.329$	0.149	0.327	V = 0.137	285	No
Governance & accountability	Q23 Support for a dedicated AI malpractice legal framework	$\chi^2(3) = 5.260$	0.154	0.327	V = 0.136	286	No
Governance & accountability	Q27 Support for standardized clinical protocols	$\chi^2(3) = 5.185$	0.159	0.327	V = 0.135	285	No
Governance & accountability	Q52 — Universities	$\chi^2(3) = 4.582$	0.205	0.398	V = 0.127	285	No
Governance & accountability	Q53 Support for international certification standards	$\chi^2(6) = 8.162$	0.226	0.415	V = 0.119	287	No
Governance & accountability	Q51 Awareness of the European AI Act	$\chi^2(3) = 4.031$	0.258	0.448	V = 0.119	287	No
Governance & accountability	Q52 — Ministry of Health	$\chi^2(3) = 3.783$	0.286	0.456	V = 0.115	285	No
Governance & accountability	Q24 — other applications	$\chi^2(3) = 3.655$	0.301	0.456	V = 0.113	287	No
Governance & accountability	Q29 — other applications	$\chi^2(3) = 3.621$	0.305	0.456	V = 0.113	286	No
Governance & accountability	Q24 — Periodic ethical evaluation of how decisions are made and communicated	$\chi^2(3) = 3.522$	0.318	0.456	V = 0.111	287	No
Governance & accountability	Q16 Preferred regulatory level for mandatory AI performance evaluation	$\chi^2(12) = 12.821$	0.382	0.525	V = 0.122	286	No

Governance & accountability	Q22 — developer responsible	$\chi^2(3) = 2.458$	0.483	0.638	V = 0.093	283	No
Governance & accountability	Q52 — Professional societies	$\chi^2(3) = 2.099$	0.552	0.701	V = 0.086	285	No
Governance & accountability	Q44 Support for legal auditing of AI algorithms	$\chi^2(3) = 1.932$	0.587	0.713	V = 0.083	282	No
Governance & accountability	Q37 Importance of an ethical oversight body	H(3) = 1.846	0.605	0.713	$\varepsilon^2 = -0.004$	287	No
Governance & accountability	Q52 — Romanian College of Dentists	$\chi^2(3) = 1.311$	0.727	0.827	V = 0.068	285	No
Governance & accountability	Q24 — Clear information to patients about the use of AI in the decision-making process	$\chi^2(3) = 1.167$	0.761	0.837	V = 0.064	287	No
Governance & accountability	Q24 — Clear allocation of responsibility between developer and clinician — who is responsible if AI makes an error?	$\chi^2(3) = 0.954$	0.812	0.865	V = 0.058	287	No
Governance & accountability	Q17 Importance of professional involvement in AI validation	H(3) = 0.370	0.946	0.976	$\varepsilon^2 = -0.009$	287	No
Governance & accountability	Q22 — clinician responsible	$\chi^2(3) = 0.093$	0.993	0.993	V = 0.018	283	No
Transparency & ethics	Q45 Support for legal obligation to inform patients	$\chi^2(6) = 16.409$	0.012	0.075	V = 0.169	286	No
Transparency & ethics	Q18 Perceived risk to patient-data confidentiality	H(3) = 7.624	0.054	0.294	$\varepsilon^2 = 0.016$	286	No
Transparency & ethics	Q19 Knowledge/application of GDPR in practice	H(3) = 6.750	0.080	0.294	$\varepsilon^2 = 0.013$	285	No
Transparency & ethics	Q49 Support for labelling automated AI applications	$\chi^2(6) = 8.690$	0.192	0.527	V = 0.123	286	No
Transparency & ethics	Q35 AI could negatively affect equity in dental care	$\chi^2(3) = 4.141$	0.247	0.543	V = 0.120	286	No
Transparency & ethics	Q20 Need for specific legislation on AI processing of biometric data	$\chi^2(3) = 2.861$	0.414	0.615	V = 0.100	287	No
Transparency & ethics	Q42 Importance of clear explanations for AI decisions	H(3) = 2.657	0.448	0.615	$\varepsilon^2 = -0.001$	287	No
Transparency & ethics	Q21 AI access to electronic patient records	$\chi^2(6) = 5.580$	0.472	0.615	V = 0.099	286	No
Transparency & ethics	Q43 Would not trust unverifiable AI recommendations	$\chi^2(3) = 1.944$	0.584	0.615	V = 0.082	286	No
Transparency & ethics	Q50 Importance of aligning legislation with EU AI Act	H(3) = 1.933	0.586	0.615	$\varepsilon^2 = -0.004$	287	No
Transparency & ethics	Q36 Support for testing AI on diverse populations	$\chi^2(3) = 1.801$	0.615	0.615	V = 0.079	287	No
Implementation	**Q46 Frequency of AI use in dental-service promotion**	H(3) = 14.483	0.002	**0.044**	$\varepsilon^2 = 0.041$	284	Yes
Implementation	Q48 — Legislation	$\chi^2(3) = 10.309$	0.016	0.153	V = 0.190	286	No
Implementation	Q28 — patient monitoring	$\chi^2(3) = 8.120$	0.044	0.276	V = 0.169	283	No
Implementation	Q40 Perceived barrier to public-sector implementation	$\chi^2(3) = 6.624$	0.085	0.282	V = 0.152	286	No
Implementation	Q28 — treatment planning	$\chi^2(3) = 6.359$	0.095	0.282	V = 0.150	283	No
Implementation	Q48 — other applications	$\chi^2(3) = 6.252$	0.100	0.282	V = 0.148	286	No
Implementation	Q28 — prosthetic design	$\chi^2(3) = 6.163$	0.104	0.282	V = 0.148	283	No
Implementation	Q41 Support for CNAS AI/digitalization funding	$\chi^2(3) = 5.237$	0.155	0.369	V = 0.135	286	No
Implementation	Q28 — diagnostic support	$\chi^2(3) = 4.914$	0.178	0.376	V = 0.132	283	No
Implementation	Q28 — patient record completion	$\chi^2(3) = 4.573$	0.206	0.376	V = 0.127	283	No
Implementation	Q48 — Standardised clinical protocols	$\chi^2(3) = 4.442$	0.217	0.376	V = 0.125	286	No
Implementation	Q28 — clinical management	$\chi^2(3) = 4.082$	0.253	0.400	V = 0.120	283	No
Implementation	Q28 — lesion interpretation	$\chi^2(3) = 2.906$	0.406	0.594	V = 0.101	283	No

Implementation	Q48 — Professional practice guidelines	$\chi^2(3) = 1.916$	0.590	0.798	V = 0.082	286	No
Implementation	Q38 Support for AI-assisted patient triage	$\chi^2(3) = 1.731$	0.630	0.798	V = 0.078	287	No
Implementation	Q39 Perceived feasibility of AI in public clinics within 5 years	H(3) = 1.533	0.675	0.801	$\varepsilon^2 = -0.005$	287	No
Implementation	Q47 Support for restricting the term AI in marketing	$\chi^2(6) = 2.377$	0.882	0.942	V = 0.064	286	No
Implementation	Q28 — histopathological analysis	$\chi^2(3) = 0.601$	0.896	0.942	V = 0.046	283	No
Implementation	Q28 — short- or long-term prognostic assessment	$\chi^2(3) = 0.393$	0.942	0.942	V = 0.037	283	No

Table S5. Complete pairwise post hoc comparison results

Pairwise comparisons for every outcome that remained significant after the domain-level FDR correction in Table S4. Binary outcomes: Fisher's exact test with Benjamini–Hochberg adjustment. Ordinal outcomes: Dunn's test with Holm adjustment. p_adj = multiplicity-adjusted p-value for the pairwise contrast. Comparisons involving the forensic physician subgroup (G3, n = 21) should be interpreted cautiously.

Item / outcome	Method	Comparison	p (raw)	p (adjusted)	Significant
Q30 Participation in AI-related courses/conferences	Fisher / BH	G1 vs. G2	<0.001	**<0.001**	*
Q30 Participation in AI-related courses/conferences	Fisher / BH	G1 vs. G3	<0.001	**0.002**	*
Q30 Participation in AI-related courses/conferences	Fisher / BH	G1 vs. G4	0.002	**0.004**	*
Q30 Participation in AI-related courses/conferences	Fisher / BH	G2 vs. G4	0.004	**0.007**	*
Q30 Participation in AI-related courses/conferences	Fisher / BH	G3 vs. G4	0.255	0.306	
Q30 Participation in AI-related courses/conferences	Fisher / BH	G2 vs. G3	0.406	0.406	
Q11 Basic knowledge of AI principles	Fisher / BH	G1 vs. G2	<0.001	**<0.001**	*
Q11 Basic knowledge of AI principles	Fisher / BH	G1 vs. G4	0.010	**0.021**	*
Q11 Basic knowledge of AI principles	Fisher / BH	G2 vs. G3	0.008	**0.021**	*
Q11 Basic knowledge of AI principles	Fisher / BH	G2 vs. G4	0.028	**0.042**	*
Q11 Basic knowledge of AI principles	Fisher / BH	G1 vs. G3	0.450	0.450	
Q11 Basic knowledge of AI principles	Fisher / BH	G3 vs. G4	0.426	0.450	
Q26 Exposure to AI-related professional guidelines	Dunn / Holm	G1 vs. G2	<0.001	**<0.001**	*
Q26 Exposure to AI-related professional guidelines	Dunn / Holm	G1 vs. G3	<0.001	**0.002**	*
Q26 Exposure to AI-related professional guidelines	Dunn / Holm	G1 vs. G4	0.011	**0.043**	*
Q26 Exposure to AI-related professional guidelines	Dunn / Holm	G3 vs. G4	0.138	0.413	
Q26 Exposure to AI-related professional guidelines	Dunn / Holm	G2 vs. G3	0.343	0.687	
Q26 Exposure to AI-related professional guidelines	Dunn / Holm	G2 vs. G4	0.410	0.687	
Q14 Familiarity with AI testing standards	Dunn / Holm	G1 vs. G2	<0.001	**<0.001**	*
Q14 Familiarity with AI testing standards	Dunn / Holm	G1 vs. G3	0.003	**0.014**	*
Q14 Familiarity with AI testing standards	Dunn / Holm	G1 vs. G4	0.135	0.361	
Q14 Familiarity with AI testing standards	Dunn / Holm	G2 vs. G4	0.090	0.361	
Q14 Familiarity with AI testing standards	Dunn / Holm	G3 vs. G4	0.100	0.361	
Q14 Familiarity with AI testing standards	Dunn / Holm	G2 vs. G3	0.653	0.653	
Q10 Familiarity with AI-based healthcare devices	Fisher / BH	G1 vs. G2	<0.001	**<0.001**	*
Q10 Familiarity with AI-based healthcare devices	Fisher / BH	G1 vs. G3	0.099	0.175	
Q10 Familiarity with AI-based healthcare devices	Fisher / BH	G1 vs. G4	0.117	0.175	
Q10 Familiarity with AI-based healthcare devices	Fisher / BH	G2 vs. G4	0.103	0.175	
Q10 Familiarity with AI-based healthcare devices	Fisher / BH	G2 vs. G3	0.421	0.505	
Q10 Familiarity with AI-based healthcare devices	Fisher / BH	G3 vs. G4	0.789	0.789	
Q13 — treatment planning	Fisher / BH	G1 vs. G3	0.002	**0.011**	*
Q13 — treatment planning	Fisher / BH	G1 vs. G2	0.010	**0.020**	*
Q13 — treatment planning	Fisher / BH	G1 vs. G4	0.008	**0.020**	*

Q13 — treatment planning	Fisher / BH	G2 vs. G3	0.168	0.253	
Q13 — treatment planning	Fisher / BH	G3 vs. G4	0.291	0.349	
Q13 — treatment planning	Fisher / BH	G2 vs. G4	0.725	0.725	
Q13 — clinical management	Fisher / BH	G1 vs. G3	<0.001	**0.001**	*
Q13 — clinical management	Fisher / BH	G1 vs. G4	0.016	**0.048**	*
Q13 — clinical management	Fisher / BH	G1 vs. G2	0.041	0.069	
Q13 — clinical management	Fisher / BH	G2 vs. G3	0.046	0.069	
Q13 — clinical management	Fisher / BH	G3 vs. G4	0.075	0.090	
Q13 — clinical management	Fisher / BH	G2 vs. G4	0.776	0.776	
Q13 — no current AI use	Fisher / BH	G1 vs. G2	0.001	**0.006**	*
Q13 — no current AI use	Fisher / BH	G1 vs. G3	0.013	**0.040**	*
Q13 — no current AI use	Fisher / BH	G2 vs. G4	0.120	0.239	
Q13 — no current AI use	Fisher / BH	G3 vs. G4	0.187	0.280	
Q13 — no current AI use	Fisher / BH	G1 vs. G4	0.404	0.484	
Q13 — no current AI use	Fisher / BH	G2 vs. G3	1.000	1.000	
Q13 — other applications	Fisher / BH	G1 vs. G2	0.004	**0.022**	*
Q13 — other applications	Fisher / BH	G1 vs. G4	0.049	0.146	
Q13 — other applications	Fisher / BH	G1 vs. G3	0.487	0.731	
Q13 — other applications	Fisher / BH	G2 vs. G3	0.416	0.731	
Q13 — other applications	Fisher / BH	G2 vs. G4	0.745	0.745	
Q13 — other applications	Fisher / BH	G3 vs. G4	0.641	0.745	
Q22 — institution responsible	Fisher / BH	G1 vs. G2	<0.001	**0.001**	*
Q22 — institution responsible	Fisher / BH	G2 vs. G3	0.002	**0.006**	*
Q22 — institution responsible	Fisher / BH	G1 vs. G4	0.026	0.052	
Q22 — institution responsible	Fisher / BH	G3 vs. G4	0.036	0.054	
Q22 — institution responsible	Fisher / BH	G1 vs. G3	0.373	0.435	
Q22 — institution responsible	Fisher / BH	G2 vs. G4	0.435	0.435	
Q33 Support for professional certification before AI use	Fisher / BH	G1 vs. G2	<0.001	**0.001**	*
Q33 Support for professional certification before AI use	Fisher / BH	G1 vs. G4	0.024	0.071	
Q33 Support for professional certification before AI use	Fisher / BH	G1 vs. G3	0.059	0.118	
Q33 Support for professional certification before AI use	Fisher / BH	G2 vs. G4	0.497	0.745	
Q33 Support for professional certification before AI use	Fisher / BH	G2 vs. G3	0.770	0.925	
Q33 Support for professional certification before AI use	Fisher / BH	G3 vs. G4	1.000	1.000	
Q15 Importance of clinical testing before AI implementation	Dunn / Holm	G2 vs. G3	0.004	**0.025**	*
Q15 Importance of clinical testing before AI implementation	Dunn / Holm	G3 vs. G4	0.032	0.159	
Q15 Importance of clinical testing before AI implementation	Dunn / Holm	G1 vs. G2	0.059	0.235	

Q15 Importance of clinical testing before AI implementation	Dunn / Holm	G1 vs. G3	0.059	0.235	
Q15 Importance of clinical testing before AI implementation	Dunn / Holm	G1 vs. G4	0.444	0.889	
Q15 Importance of clinical testing before AI implementation	Dunn / Holm	G2 vs. G4	0.472	0.889	
Q46 Frequency of AI use in dental-service promotion	Dunn / Holm	G1 vs. G4	<0.001	**0.003**	*
Q46 Frequency of AI use in dental-service promotion	Dunn / Holm	G1 vs. G2	0.030	0.150	
Q46 Frequency of AI use in dental-service promotion	Dunn / Holm	G1 vs. G3	0.157	0.526	
Q46 Frequency of AI use in dental-service promotion	Dunn / Holm	G2 vs. G4	0.132	0.526	
Q46 Frequency of AI use in dental-service promotion	Dunn / Holm	G3 vs. G4	0.303	0.605	
Q46 Frequency of AI use in dental-service promotion	Dunn / Holm	G2 vs. G3	0.946	0.946	

Table S6. Means and standard deviations for the 1–10 ordinal outcomes

Descriptive statistics for the ordinal (1–10) questionnaire items analyzed as ordinal variables in the inferential comparisons. Data are shown as valid n, missing n, mean ± standard deviation, median, interquartile range (IQR, P25–P75), and range. Available-case analysis.

Item	Variable	Valid n	Missing n	Mean ± SD	Median	IQR (P25–P75)	Min–Max
Q14	Familiarity with AI testing standards	287	0	3.34 ± 2.78	2.0	1-5	1-10
Q15	Importance of clinical testing before AI implementation	287	0	8.88 ± 2.16	10.0	9-10	1-10
Q17	Importance of professional involvement in AI validation	287	0	9.05 ± 1.63	10.0	8-10	1-10
Q18	Perceived risk to patient-data confidentiality	286	1	5.00 ± 3.00	5.0	2-7	1-10
Q19	Knowledge/application of GDPR in current practice	285	2	6.20 ± 2.56	6.0	5-8	1-10
Q26	Exposure to AI-related professional guidelines	285	2	2.46 ± 2.22	1.0	1-3	1-10
Q31	Importance of introducing AI into undergraduate curricula	287	0	7.32 ± 2.66	8.0	5-10	1-10
Q37	Importance of an independent ethical oversight body for healthcare AI	287	0	8.33 ± 2.51	10.0	7-10	1-10
Q39	Perceived feasibility of AI implementation in public dental clinics within five years	287	0	5.55 ± 2.85	5.0	3-8	1-10
Q42	Importance of clear explanations for AI decisions	287	0	8.90 ± 1.90	10.0	8-10	1-10
Q46	Frequency of AI use in dental-service promotion	284	3	5.45 ± 2.95	5.0	3-8	1-10
Q50	Importance of aligning Romanian legislation with the EU AI Act	287	0	8.75 ± 2.03	10.0	8-10	1-10

Table S7. Exploratory psychometric evaluation of the questionnaire

This supplementary table provides additional methodological and diagnostic details for the exploratory psychometric analysis reported in Section 3.4 and Table 5 of the main manuscript. The factor structure is preliminary and the factor labels should be interpreted as descriptive empirical groupings rather than independently validated questionnaire subscales.

Panel A. Item selection and analytic approach

Items analysed (28). Exploratory psychometric analyses were conducted on 28 questionnaire items considered suitable for analysis as ordinal or binary indicators. Items were eligible when they used an ordinal (1–10) or binary response format and measured a conceptually relevant construct. Purely nominal multiple-response items (e.g., lists of applications, authorities, or safeguards from which respondents could select several options) and open-text items were not included in the psychometric analysis. Response concentration was considered during psychometric assessment, and highly skewed items were interpreted cautiously because of their reduced discriminating variance. Conceptually reverse-worded items were recoded so that higher scores denoted a higher level of the intended construct prior to analysis.

Analytic approach. Because the eligible items were binary and ordinal rather than continuous, associations among items were quantified using a **polychoric and tetrachoric correlation matrix** (polychoric correlations for pairs involving ordinal items and tetrachoric correlations for pairs of binary items). Sampling adequacy was assessed with the Kaiser–Meyer–Olkin (KMO) measure and Bartlett's test of sphericity. Factors were extracted by **principal-axis factoring** and rotated using an **oblique (direct oblimin) rotation**, as the underlying dimensions were expected to be correlated. The number of factors was determined by parallel analysis, supported by the scree plot and conceptual interpretability. The four-factor solution identified by EFA was then examined by a preliminary confirmatory factor analysis (CFA) estimated with a **robust diagonally weighted least squares (DWLS) estimator**, appropriate for ordinal and binary indicators.

Panel B. Sampling adequacy and factorability

Statistic	Value	Interpretation
Kaiser–Meyer–Olkin measure	0.695	Acceptable sampling adequacy
Bartlett's test of sphericity	$\chi^2(378) = 1117.83$, $p < 0.001$	Correlation matrix suitable for factoring
Number of items	28	Ordinal and binary indicators
Factor-retention criterion	Parallel analysis	Four factors retained

Note. Values as reported in Section 3.4 of the main manuscript. KMO ≥ 0.60 is considered acceptable for factor analysis; a significant Bartlett's test indicates that the correlation matrix differs from an identity matrix and is therefore factorable.

Panel C. Four-factor structure and internal consistency

Factor (descriptive label)	Items	No.	Cronbach's α	McDonald's ω
1. Governance and accountability safeguards	Q20, Q23, Q25, Q27, Q33, Q36, Q37, Q44	8	0.878	0.880
2. AI literacy and regulatory awareness	Q10, Q11, Q14, Q26, Q30, Q34, Q51	7	0.844	0.854
3. Public-sector implementation attitudes, equity, and perceived barriers	Q18, Q35, Q38, Q39, Q40, Q41, Q43	7	0.693	0.696
4. Clinical validation, explainability, and professional preparedness	Q15, Q17, Q19, Q31, Q42, Q50	6	0.703	0.720

Note. α = Cronbach's alpha; ω = McDonald's omega, as reported in Table 5 of the main manuscript. Factor-specific estimates ranged from α = 0.693 to 0.878 and from ω = 0.696 to 0.880. Reliability was highest for the governance and accountability factor and the AI literacy and regulatory awareness factor; the clinical validation, explainability, and professional preparedness factor showed acceptable internal consistency; and the

public-sector implementation, equity, and perceived-barriers factor showed comparatively lower, borderline internal consistency. Factor labels are descriptive interpretations of the empirical item groupings and should not be treated as independently validated subscales.

Panel D. Preliminary confirmatory factor analysis

The four-factor solution was tested by a preliminary CFA estimated with a robust DWLS estimator. Because the exploratory and confirmatory analyses used the same analytical sample, the CFA is a preliminary assessment of structural fit, not an independent validation.

Fit index	Value	Interpretation
χ^2 (df)	380.146 (246)	Model chi-square
χ^2/df	1.55	Acceptable (< 2)
CFI	0.885	Below the 0.90 incremental-fit threshold
TLI	0.871	Below the 0.90 incremental-fit threshold
RMSEA	0.045	Good absolute fit (< 0.08)
SRMR	0.076	Acceptable residual fit (< 0.08)

Note. Values as reported in Section 3.4 of the main manuscript. The RMSEA, SRMR, and χ^2/df indicated generally acceptable absolute and residual fit, whereas the CFI and TLI remained below commonly used thresholds for incremental fit. Accordingly, the four-factor structure is regarded as preliminary rather than independently validated.

Panel E. Inter-factor correlations

The four factors were modelled as correlated (oblique solution). In the confirmatory model, the latent factors were only weakly inter-correlated: the only statistically significant correlation was a small negative association between the governance and accountability factor and the AI literacy and regulatory awareness factor ($r \approx -0.20$, $p < 0.05$); all remaining inter-factor correlations were small and non-significant ($|r| \leq 0.17$). This pattern indicates that the four dimensions capture largely distinct aspects of stakeholder perception, consistent with an oblique but weakly correlated structure.

Note. Because the factors were weakly correlated, the oblique and orthogonal solutions yielded substantively similar interpretations.

Factors	*r*	*p*
F1–F2	−0.199	0.019
F1–F3	0.165	0.082
F1–F4	0.163	0.112
F2–F3	0.150	0.088
F2–F4	−0.042	0.647
F3–F4	−0.148	0.169

Only the correlation between Factor 1 (governance and accountability safeguards) and Factor 2 (AI literacy and regulatory awareness) reached statistical significance; the remaining inter-factor correlations were small and non-significant, consistent with an oblique but weakly correlated structure.

Panel F. Interpretation and limitations

The four-factor solution is interpretable as representing governance and accountability safeguards; AI literacy and regulatory awareness; public-sector implementation attitudes, equity, and perceived barriers; and clinical validation, explainability, and professional preparedness. These factors are descriptive groupings of conceptually related items rather than psychometrically validated subscales. Three considerations constrain their status: the exploratory and confirmatory analyses were conducted in the same sample, so the confirmatory step is not independent; the incremental fit indices (CFI, TLI) fell below conventional thresholds; and one factor (public-sector implementation, equity, and perceived barriers) showed borderline internal consistency. Accordingly, no factor scores were computed or used in the inferential analyses reported in the main text, which are based on individual items. External validation in an independent sample — preferably with a prespecified factor structure and assessment of measurement invariance across stakeholder groups — is required before the instrument can be used to derive scored subscales.

Supplementary Table S7A. Item-level rationale for inclusion in the exploratory psychometric analysis (EFA)

The table lists all 53 questionnaire items and identifies the 28 items included in the exploratory psychometric analysis (EFa) reported in the main manuscript (Table 5). Selection was based on conceptual relevance and suitability as binary or ordered indicators for exploratory factor analysis. Administrative, demographic, nominal multi-category, and multiple-response items were excluded. Response concentration was considered during psychometric assessment but was not used as an automatic exclusion criterion; highly skewed retained items were interpreted cautiously. Factor allocation follows the four-factor structure reported in Table 5. Q19 and Q40 were retained but should be interpreted cautiously because their wording combines more than one conceptual element. Q46 was excluded despite its ordinal response scale because it was treated analytically as a standalone current-use/marketing outcome rather than as an indicator of the exploratory latent structure.

Item	Short description	Response format	Included in psychometric analysis	Rationale for inclusion / exclusion	Final factor allocation
Q1	E-mail address	Administrative	No	Administrative/contact item; not a substantive AI-related construct.	—
Q2	Consent to participate	Administrative/ethics	No	Research-consent item; not a substantive questionnaire construct.	—
Q3	Consent to research-data processing	Administrative/ethics	No	Data-processing/consent item; not a substantive AI-related construct.	—
Q4	Age	Demographic	No	Sociodemographic descriptor used for sample characterization, not a latent AI-related indicator.	—
Q5	Gender	Demographic	No	Sociodemographic descriptor, not a latent AI-related indicator.	—
Q6	Professional category / specialty	Classification	No	Defines stakeholder/professional group; used for stratification rather than psychometric measurement.	—
Q7	University faculty status	Classification	No	Professional/academic classification variable, not a latent questionnaire indicator.	—
Q8	Region of professional activity	Demographic/geographic	No	Geographic descriptor used for sample characterization, not psychometric measurement.	—
Q9	Urban/rural professional environment	Demographic/contextual	No	Practice-setting descriptor, not a latent AI-related indicator.	—
**Q10**	**Familiarity with AI-based healthcare devices**	**Binary**	**Yes**	**Conceptually relevant binary indicator of baseline AI familiarity; suitable for mixed binary/ordinal factor analysis.**	**F2 – AI literacy and regulatory awareness**
**Q11**	**Basic knowledge of AI principles**	**Binary**	**Yes**	**Direct binary indicator of self-reported foundational AI knowledge and literacy.**	**F2 – AI literacy and regulatory awareness**

Item	Short description	Response format	Included in psychometric analysis	Rationale for inclusion / exclusion	Final factor allocation
Q12	Perceived advantages of AI in medicine	Multiple response	No	Multiple-response item with several non-independent option-level outcomes; not a single latent indicator.	—
Q13	Current professional uses of AI	Multiple response	No	Multiple-response use-case item with several option-level outcomes and substantial role-related non-response; analyzed separately rather than as a single psychometric indicator.	—
**Q14**	**Familiarity with AI testing standards**	**Ordinal (1–10)**	**Yes**	**Ordered indicator of awareness of AI testing/validation standards; conceptually relevant to AI and regulatory literacy.**	**F2 – AI literacy and regulatory awareness**
**Q15**	**Importance of clinical testing before AI implementation**	**Ordinal (1–10)**	**Yes**	**Ordered attitude indicator directly addressing clinical validation and pre-implementation safeguards.**	**F4 – Clinical validation, explainability, and professional preparedness**
Q16	Preferred regulatory level for mandatory AI performance evaluation	Nominal single-choice	No	Unordered categorical preference (European, national, combined, other); categories do not form an ordinal latent continuum.	—
**Q17**	**Importance of professional involvement in AI validation**	**Ordinal (1–10)**	**Yes**	**Ordered indicator of professional involvement and validation expectations.**	**F4 – Clinical validation, explainability, and professional preparedness**
**Q18**	**Perceived risk to patient-data confidentiality**	**Ordinal (1–10)**	**Yes**	**Ordered indicator of perceived privacy/confidentiality risk relevant to implementation attitudes and safeguards.**	**F3 – Public-sector implementation attitudes, equity, and perceived barriers**
**Q19**	**Knowledge and application of GDPR in current practice**	**Ordinal (1–10)**	**Yes**	**Ordered indicator of perceived regulatory knowledge/application; retained as conceptually relevant, while interpreted cautiously because the wording combines knowledge and application.**	**F4 – Clinical validation, explainability, and professional preparedness**
**Q20**	**Support for biometric-data legislation**	**Binary**	**Yes**	**Binary governance-safeguard indicator. Retained despite response concentration because skewness was considered during interpretation rather than used as an automatic exclusion criterion.**	**F1 – Governance and accountability safeguards**
Q21	AI access to electronic health records for treatment suggestions	Nominal single-choice	No	Unordered categorical item combining different access/consent positions; not suitable as a single ordered or binary latent indicator.	—
Q22	Responsibility for an incorrect AI-generated diagnosis	Multiple response	No	Multiple-response attribution item; selections are not mutually exclusive and represent distinct actors rather than one latent indicator.	—
**Q23**	**Support for a dedicated AI malpractice legal framework**	**Binary**	**Yes**	**Binary indicator of support for a specific accountability/legal safeguard.**	**F1 – Governance and accountability safeguards**
Q24	Measures ensuring traceability of AI-assisted decisions	Multiple response	No	Composite multiple-response item covering several distinct safeguards; option-level selections were analyzed separately.	—
**Q25**	**Would refrain from AI use if liability unclear**	**Binary**	**Yes**	**Binary indicator of legal-certainty concerns and risk-related restraint in AI use.**	**F1 – Governance and accountability safeguards**
**Q26**	**Exposure to AI-related professional guidelines**	**Ordinal (1–10)**	**Yes**	**Ordered indicator of exposure to professional AI guidance and regulatory/professional awareness.**	**F2 – AI literacy and regulatory awareness**

Item	Short description	Response format	Included in psychometric analysis	Rationale for inclusion / exclusion	Final factor allocation
**Q27**	**Support for standardized clinical protocols**	**Binary**	**Yes**	**Binary governance indicator reflecting support for standardized clinical safeguards; retained despite response concentration and interpreted cautiously.**	**F1 – Governance and accountability safeguards**
Q28	Dental interventions most suitable for AI integration	Multiple response	No	Multiple-response prioritization item representing distinct clinical use cases rather than a single latent construct.	—
Q29	Stakeholders responsible for national dental AI guidelines	Multiple response	No	Multiple-response institutional-responsibility item; options represent distinct actors and are not a single psychometric indicator.	—
**Q30**	**Previous participation in AI-related courses/conferences**	**Binary**	**Yes**	**Binary indicator of prior AI-related educational exposure; conceptually relevant to professional AI literacy/preparedness.**	**F2 – AI literacy and regulatory awareness**
**Q31**	**Importance of introducing AI into undergraduate curricula**	**Ordinal (1–10)**	**Yes**	**Ordered indicator of support for formal AI education and professional preparedness.**	**F4 – Clinical validation, explainability, and professional preparedness**
Q32	Preferred educational format	Nominal preference item	No	Unordered preference categories (e.g., workshops, software training, webinars); not an ordinal or binary latent indicator.	—
**Q33**	**Professional certification before AI use**	**Binary**	**Yes**	**Binary indicator of competency assurance and professional-governance safeguards.**	**F1 – Governance and accountability safeguards**
**Q34**	**Familiarity with algorithmic bias**	**Binary**	**Yes**	**Binary indicator of AI literacy concerning bias and fairness concepts.**	**F2 – AI literacy and regulatory awareness**
**Q35**	**AI could negatively affect equity in dental care**	**Binary**	**Yes**	**Binary indicator of perceived equity risk relevant to implementation and fairness concerns.**	**F3 – Public-sector implementation attitudes, equity, and perceived barriers**
**Q36**	**Support for testing AI on diverse populations**	**Binary**	**Yes**	**Binary safeguard/equity indicator; retained despite marked response concentration because it was conceptually central and skewness was handled interpretively.**	**F1 – Governance and accountability safeguards**
**Q37**	**Importance of an independent ethical oversight body for healthcare AI**	**Ordinal (1–10)**	**Yes**	**Ordered governance/oversight indicator suitable for factor analysis.**	**F1 – Governance and accountability safeguards**
**Q38**	**Support for AI-assisted patient triage**	**Binary**	**Yes**	**Binary indicator of perceived public-sector clinical utility and implementation attitude.**	**F3 – Public-sector implementation attitudes, equity, and perceived barriers**
**Q39**	**Perceived feasibility of AI in public clinics within 5 years**	**Ordinal (1–10)**	**Yes**	**Ordered indicator of perceived near-term public-sector implementation realism.**	**F3 – Public-sector implementation attitudes, equity, and perceived barriers**
**Q40**	**Perceived barrier to public-sector implementation**	**Binary**	**Yes**	**Binary implementation-barrier indicator; retained as conceptually relevant, while interpreted cautiously because the item combines several barrier dimensions.**	**F3 – Public-sector implementation attitudes, equity, and perceived barriers**

Item	Short description	Response format	Included in psychometric analysis	Rationale for inclusion / exclusion	Final factor allocation
**Q41**	**Support for CNAS AI/digitalization funding**	**Binary**	**Yes**	**Binary policy/implementation indicator reflecting support for dedicated public-sector resources.**	**F3 – Public-sector implementation attitudes, equity, and perceived barriers**
**Q42**	**Importance of clear explanations for AI decisions**	**Ordinal (1–10)**	**Yes**	**Ordered explainability indicator directly relevant to professional judgment and AI governance.**	**F4 – Clinical validation, explainability, and professional preparedness**
**Q43**	**Would not trust unverifiable AI recommendations**	**Binary**	**Yes**	**Binary indicator of trust under opacity/limited explainability; retained despite response concentration and interpreted cautiously.**	**F3 – Public-sector implementation attitudes, equity, and perceived barriers**
**Q44**	**Support for legal auditing of AI algorithms**	**Binary**	**Yes**	**Binary auditability/accountability safeguard indicator; retained despite response concentration because of strong conceptual relevance.**	**F1 – Governance and accountability safeguards**
Q45	Legal obligation to inform patients when AI is used in treatment decisions	Nominal categorical	No	Categorical response structure with more than two unordered categories; analyzed as a standalone outcome rather than as a binary/ordinal latent indicator.	—
Q46	Perceived current frequency of AI use in dental-service promotion	Ordinal (1–10)	No	Although ordinal, it was treated as a distinct current-use/marketing outcome rather than as an indicator of the exploratory latent questionnaire structure.	—
Q47	Support for restricting the term AI in marketing	Nominal categorical	No	Categorical policy-attitude response with unordered categories; not used as a single ordinal/binary latent indicator.	—
Q48	Preferred regulation to prevent misleading AI-related advertising	Multiple response	No	Multiple-response governance item with distinct regulatory options; not a single latent indicator.	—
Q49	Clear labelling of automated systems used in patient communication	Nominal categorical	No	Categorical disclosure item with unordered response categories; analyzed descriptively/inferentially as a standalone outcome.	—
**Q50**	**Importance of aligning Romanian legislation with the EU AI Act**	**Ordinal (1–10)**	**Yes**	**Ordered regulatory-alignment indicator relevant to governance, explainability, and professional preparedness.**	**F4 – Clinical validation, explainability, and professional preparedness**
**Q51**	**Awareness of the EU Artificial Intelligence Act**	**Binary**	**Yes**	**Binary indicator of regulatory awareness and literacy.**	**F2 – AI literacy and regulatory awareness**
Q52	Romanian institutions that should implement AI regulation in healthcare	Multiple response	No	Multiple-response institutional-governance item; each selected institution represents a separate option-level outcome.	—
Q53	Support for international certification standards	Nominal categorical	No	Categorical standardization/certification item with unordered response categories; analyzed as a standalone outcome rather than a latent indicator.	—

Table S8. Sensitivity analyses

Sensitivity analysis	Rationale	Items re-examined	Effect on primary findings
Exclusion of the healthcare stakeholder group (G4)	G4 is heterogeneous (mixed non-dental healthcare roles); its removal tests whether between-group differences depend on this composite group.	All FDR-significant items in Table S4	**Robust (12/13 retained).** Of the 13 findings significant in the full sample, 12 remained significant after FDR correction (N = 244). Effect sizes for the core literacy, education, accountability, and Q13 application items were unchanged or slightly strengthened (e.g., Q11, Q26, Q30 $p_{FDR} < 0.001$; Q22 institution $p_{FDR} = 0.002$; Q13 clinical management $p_{FDR} = 0.002$). The single exception was frequency of AI use in dental-service promotion (Q46), which lost significance (full sample $H(3) = 14.483$, $p_{FDR} = 0.044$; without G4 $H(2) = 5.864$, raw $p = 0.053$), consistent with this difference being driven by the G1–G4 contrast. No new domain-level conclusions emerged. The principal findings are therefore robust to exclusion of the heterogeneous healthcare-stakeholder group.
Exclusion of the forensic physician subgroup (G3, n = 21)	G3 is small; pairwise contrasts involving it are less stable. Removal tests whether conclusions rest on this subgroup.	Q15, Q22 (institution), and all items with a significant G3 contrast	**Robust (12/13 retained).** Of the 13 findings significant in the full sample, 12 remained significant after FDR correction (N = 266). All literacy, education, accountability, certification, and Q13 application items retained significance with comparable effect sizes (e.g., Q30, Q11 $p_{FDR} < 0.001$; Q22 institution $p_{FDR} = 0.005$; Q33 $p_{FDR} = 0.005$; Q46 $p_{FDR} = 0.017$). The single exception was the importance of clinical testing before AI implementation (Q15), which lost significance (full sample $H(3) = 13.159$, $p_{FDR} = 0.047$; without G3 $H(2) = 5.081$, raw $p = 0.079$) — expected, because its only surviving pairwise contrast in the full sample was G2 versus G3. Conclusions involving the small forensic-physician subgroup should thus be regarded as exploratory, whereas all other between-group differences are stable.
Inclusion of participants with item-level missing data	Available-case analysis excludes item non-responders; this check tests sensitivity to the missing-data handling, notably the 20.6% non-response on Q13.	Q13 sub-items and all Table S4 items with missing data	**Direction unchanged; strongest findings retained.** The primary analyses used available cases (Q13 valid n = 228). In a re-analysis over the full group-classified sample

			(N = 287), in which non-response to the multiple-response Q13 item was treated as non-selection of an application, the two strongest Q13 findings were retained and strengthened (clinical management $\chi^2(3) = 26.691$, $p < 0.001$, $V = 0.305$; treatment planning $\chi^2(3) = 26.666$, $p < 0.001$, $V = 0.305$). The weaker Q13 contrasts attenuated (other applications $\chi^2(3) = 5.970$, $p = 0.113$; no current AI use $\chi^2(3) = 8.726$, $p = 0.033$), consistent with the differential, group-related non-response documented in Section 4.7 ($\chi^2(3) = 33.247$, $p < 0.001$). The direction of all Q13 effects was unchanged, and no finding reversed. This confirms that the principal Q13 conclusions do not depend on the missing-data handling, while reinforcing that the weaker Q13 contrasts warrant cautious interpretation.
